# Stevia-derived sweeteners in reduced-sugar foods: formulation strategies, sensory optimization, nutritional evidence, and safety perspectives

**DOI:** 10.3389/fnut.2026.1894194

**Published:** 2026-08-05

**Authors:** Lisha Wang, Tianying Chang, Wenkang Jia, Jiahui Chen, Le Li, Tianpeng Zhang, Bo Wang, Chenxuan Dong, Yingzi Cui, Xing Liao

**Affiliations:** 1 College of Traditional Chinese Medicine, Changchun University of Chinese Medicine, Changchun, China; 2 EBM Office, The Affiliated Hospital to Changchun University of Chinese Medicine, Changchun, China; 3 Collaboration Network of the China Center for Evidence-Based Traditional Chinese Medicine (On Probation), Changchun, China; 4 GCP Office, The Affiliated Hospital to Changchun University of Chinese Medicine, Changchun, China; 5 Institute of Basic Research in Clinical Medicine, China Academy of Chinese Medical Sciences, Beijing, China

**Keywords:** alternative sweeteners, food reformulation, functional foods, safety assessment, sensory optimization, *Stevia rebaudiana*, steviol glycosides, sugar reduction

## Abstract

**Background:**

Excessive intake of added sugars is an important contributor to obesity, type 2 diabetes mellitus (T2DM), dental caries, cardiovascular disease, and other metabolism-related disorders in humans. Stevia-derived sweeteners, particularly high-purity steviol glycosides (SGs), have been widely used in reduced-sugar and sugar-free foods because of their high sweetness intensity, low energy contribution, and plant-derived origin. However, their broader application remains limited by bitterness, lingering sweetness, licorice-like or herbal aftertaste, thin mouthfeel, and food-matrix dependence.

**Methods:**

This narrative review critically summarizes the current evidence on stevia-derived sweeteners, focusing on taste perception and sensory-related signaling, sensory optimization, nutritional and metabolic evidence, food applications, safety, and regulatory requirements. This review also discusses the challenges and prospects of developing reduced-sugar foods.

**Results:**

Current evidence indicates that high-purity SGs are important sucrose substitutes; however, their application performance is influenced by glycoside structure, sweet and bitter taste receptor interactions, central sensory processing, and food-matrix characteristics. Glycoside blending, enzymatic conversion, microbial fermentation, biocatalysis, structural modification, and food-matrix optimization can improve the sensory quality, acceptability, mouthfeel, and formulation compatibility of SGs. Nutritional and metabolic evidence suggests that SGs mainly reduce sugar and energy intake by substituting sucrose. At the same time, their potential roles in glycemic regulation, body weight management, gut health, antioxidant activity, inflammation, and cardiometabolic health require further validation in human studies.

**Conclusion:**

By integrating sensory mechanisms, food formulation, nutritional evidence, safety assessment, and regulatory perspectives, this review provides a scientific basis for the rational use of stevia-derived sweeteners, especially SGs, in functional reduced-sugar foods. Future research should prioritize sensory optimization in real food matrices, long-term human studies on metabolic and dietary outcomes, and exposure assessment in vulnerable or high-consumption populations to support the safe, effective, and regulatory-compliant application.

## Graphical Abstract

**Figure:**
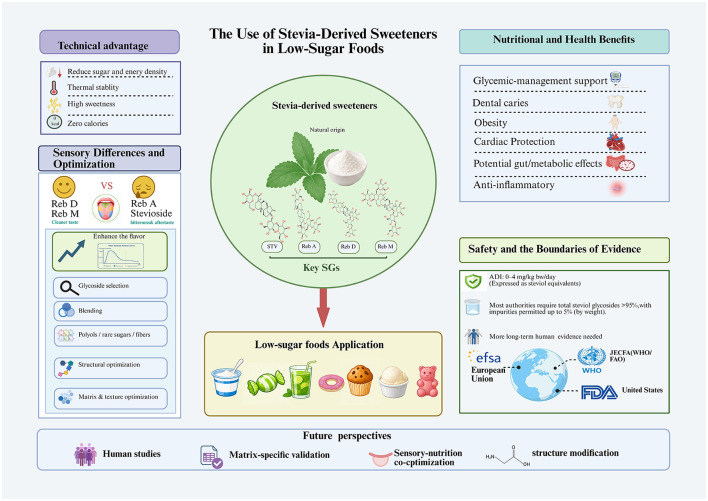
Created with BioRender. Lisha W. (2026) https://BioRender.com/2xwat8f.

## 1 Introduction

High-sugar diets, particularly excessive sucrose intake, are recognized as important contributors to the increasing global burden of obesity, type 2 diabetes mellitus (T2DM), dental caries, cardiovascular disease, and other metabolic disorders, placing substantial pressure on public health and healthcare systems (1–4). Although the World Health Organization (WHO) recommends limiting free sugar intake to less than 10% of the total daily energy intake, with a further reduction to below 5% where feasible (4), sugar intake remains above the recommended levels in many countries. For example, sugar intake accounts for more than 13 and 12% of the total energy intake in the United States and the United Kingdom, respectively (5). Observational evidence cited in dietary guidelines suggests that individuals consuming 25% or more of their daily energy from added sugars have a higher risk of cardiovascular disease mortality than those consuming less than 10%, supporting recommendations to limit added sugar intake (6). Therefore, reducing the added sugar in foods and beverages has become an important public health goal and a major driver for the development of low- and no-calorie sweeteners (LNCSs) and natural sugar substitutes (7).

Non-nutritive sweeteners (NNSs) can be used to reduce the sugar and energy content of foods. However, concerns remain regarding their sensory quality, consumer acceptance, and potential long-term health effects (7–12). Therefore, the development of reduced-sugar foods should not be limited to energy replacement, but should also consider sweetness quality, food matrix compatibility, nutritional appropriateness, and long-term consumer acceptance, thereby supporting more sustainable dietary improvement and potential benefits for cardiometabolic health management (6).

In response to this demand for reduced-sugar food development, stevia-derived sweeteners have attracted sustained attention as plant-derived sugar substitutes in low-sugar and sugar-free food applications (13). Steviol glycosides (SGs) are the major sweet-tasting constituents of *Stevia rebaudiana* Bertoni leaves, with a sweetness intensity of approximately 200–450 times that of sucrose (13, 14). In addition to SGs, stevia leaves may contain non-glycosidic constituents, including polyphenols, polysaccharides, amino acids, fatty acids, and volatile compounds, which may be associated with sensory modulation and potential nutritional and metabolic effects (14–18).

SGs provide high-intensity sweetness but are often associated with bitterness, lingering sweetness, licorice-like or herbal aftertastes, and other sensory drawbacks, making it difficult for them to fully reproduce the overall sensory quality of sucrose (19). Moreover, sucrose contributes not only sweetness but also multiple technological functions in foods, including bulking, viscosity modulation, texture formation, crystallization behavior, browning reactions, provision of a fermentation substrate, and matrix stabilization (20, 21). Therefore, effective sugar reduction using stevia-derived sweeteners is not simply a matter of sweetness replacement, but an integrated development challenge involving raw material characteristics, glycoside composition, sensory optimization, food matrix compatibility, potential nutritional and metabolic effects, safety assessment, and regulatory compliance.

Existing studies have examined stevia and its SGs from multiple perspectives, including phytochemical composition, extraction and purification, glycoside structure and sensory properties, food applications, safety assessment, potential health effects, and processing technologies (14, 22–25). However, these studies have largely focused on individual dimensions, and integrative research remains limited within the framework of reduced-sugar food development. In particular, few reviews have linked the botanical and chemical characteristics of stevia with taste and central reward mechanisms, sensory optimization, formulation strategies, potential nutritional and metabolic effects, food applications, safety assessment, and regulatory requirements. To address this research gap, this review critically integrates evidence on stevia-derived sweeteners, particularly SGs, in the development of reduced-sugar foods, focusing on their application basis, major challenges, and future directions. By doing so, it aims to provide scientific support for formulation design, assessment of nutritional potential, safety evaluation, regulatory-compliant application, and translation into real food systems.

## 2 Methods

### 2.1 Literature search strategy

This study adopted a narrative review approach to critically summarize current evidence on stevia and its major steviol glycosides (SGs) in the development of reduced-sugar foods. The literature search focused on the botanical origin and chemical composition of stevia; sweetness characteristics; sensory limitations; extraction and modification technologies; formulation applications; nutritional and metabolic effects; gut microbiota-related effects; safety evaluation; regulatory status; and comparisons with other low- and no-calorie sweeteners (LNCSs). The search covered the period from database inception to June 28, 2026, with priority given to studies published within the past decade.

### 2.2 Databases and search terms

The literature search was mainly conducted in Web of Science, PubMed, ScienceDirect, and Google Scholar. In addition, to include evidence from food science, nutrition, safety assessment, and regulation, official websites and regulatory documents from relevant international organizations and regulatory agencies were also searched, including the World Health Organization (WHO), the Joint FAO/WHO Expert Committee on Food Additives (JECFA), the U.S. Food and Drug Administration (FDA), the European Food Safety Authority (EFSA), and relevant Chinese food safety standards. Product information from e-commerce platforms was used only to illustrate current application scenarios and was not considered scientific evidence. The main English search terms were combined using Boolean operators (“AND” and “OR”) and included terms related to stevia and steviol glycosides, sweeteners, food formulation, sensory properties, safety, and regulation. The search strategy included combinations such as (“*Stevia rebaudiana*” OR stevia OR “steviol glycosides” OR stevioside OR “rebaudioside A” OR “rebaudioside D” OR “rebaudioside M” OR “Reb A” OR “Reb D” OR “Reb M”) AND (“natural sweetener” OR “non-nutritive sweetener” OR “low-calorie sweetener” OR “sugar substitute” OR “sugar reduction”) AND (“food formulation” OR “sensory profile” OR “bitter aftertaste” OR “taste receptor” OR “T1R2/T1R3” OR TAS2R OR safety OR toxicology OR “acceptable daily intake” OR “regulatory status”).

### 2.3 Inclusion and exclusion criteria

Eligible literature included original studies, randomized controlled trials, systematic reviews, meta-analyses, authoritative regulatory documents, food standards, and relevant review articles. Priority was given to studies published within the past decade and to literature addressing the sensory properties, formulation applications, nutritional and metabolic effects, safety assessment, regulatory status, and applications of stevia-derived sweeteners and SGs in reduced-sugar food matrices. Literature was excluded if it was not directly related to food applications, nutritional metabolism, sensory properties, safety evaluation, or regulatory assessment. Duplicate records, promotional materials, unsupported health claims, unverifiable market-size information, and studies with insufficient methodological or data reporting were also excluded.

### 2.4 Data extraction

Relevant information was extracted from the included literature and narratively synthesized, including the main constituents of stevia, sweetness and aftertaste characteristics, extraction and modification technologies, food application scenarios, nutritional and metabolic effects, safety evaluation, regulatory status, and comparisons with other sweeteners.

### 2.5 Chemical structure visualization

Chemical structure information for representative SGs, including stevioside (STV), rebaudioside A (Reb A), rebaudioside D (Reb D), rebaudioside M (Reb M), and their aglycone steviol, was obtained from ChemSpider (https://www.chemspider.com). These structures were used for chemical visualization and comparison of representative SGs.

## 3 Terminology

To avoid conceptual ambiguity, this review distinguishes among stevia, stevia leaves, stevia extracts, high-purity SGs, and commercial stevia-based sweetener blends. In this review, “stevia” is used as a broad umbrella term to refer to *Stevia rebaudiana*, its derived sweetening materials, and related applications. When discussing chemical composition, biological activity, sensory properties, safety, or regulatory status, more specific terminology is used whenever appropriate. “Stevia leaves” refers to the leaf material of *Stevia rebaudiana*, which serves as the primary botanical source of SGs and other phytochemical constituents. “Stevia extracts” refers to minimally purified extracts obtained from stevia leaves, which contain SGs together with non-glycosidic phytochemicals, including flavonoids, phenolic acids, essential oils, proteins, and other bioactive constituents (14, 25). “High-purity SGs” refers to purified sweetening ingredients that comply with applicable regulatory specifications, typically containing at least 95% total SGs on a dry-weight basis. Representative SGs include STV, Reb A, Reb D, and Reb M, which are the principal sweetening compounds approved for food applications (26, 27). “Commercial stevia-based sweetener blends” refers to formulated sweetener products in which high-purity SGs are combined with other sweeteners, such as erythritol, maltitol, dietary fibers, rare sugars, or other bulking agents (25, 28).

In addition, this review distinguishes among LNCSs, NNSs, and non-sugar sweeteners (NSSs) (29). The term LNCSs is used broadly in food science and nutrition to describe sweeteners that provide little or no energy (30). The term NNSs refers to sweeteners with negligible nutritive contribution. The term NSSs is primarily used in public health, guideline, or regulatory contexts, particularly in relation to WHO terminology (31).

## 4 Botanical origin, composition, and historical use of stevia

### 4.1 Botany, historical use, and distribution

The genus *Stevia* comprises approximately 268 species (32). It is mainly distributed from the southern United States to Argentina, extending through Central America to Colombia and southward to the Andes (33). Although the genus contains many species, only *Stevia rebaudiana* and *Stevia phlebophylla* have been reported to produce SGs, with *S. phlebophylla* containing relatively low levels of these compounds (13). Therefore, this review focuses primarily on *Stevia rebaudiana* Bertoni, a species with the greatest relevance to food applications.

**Figure 1:**
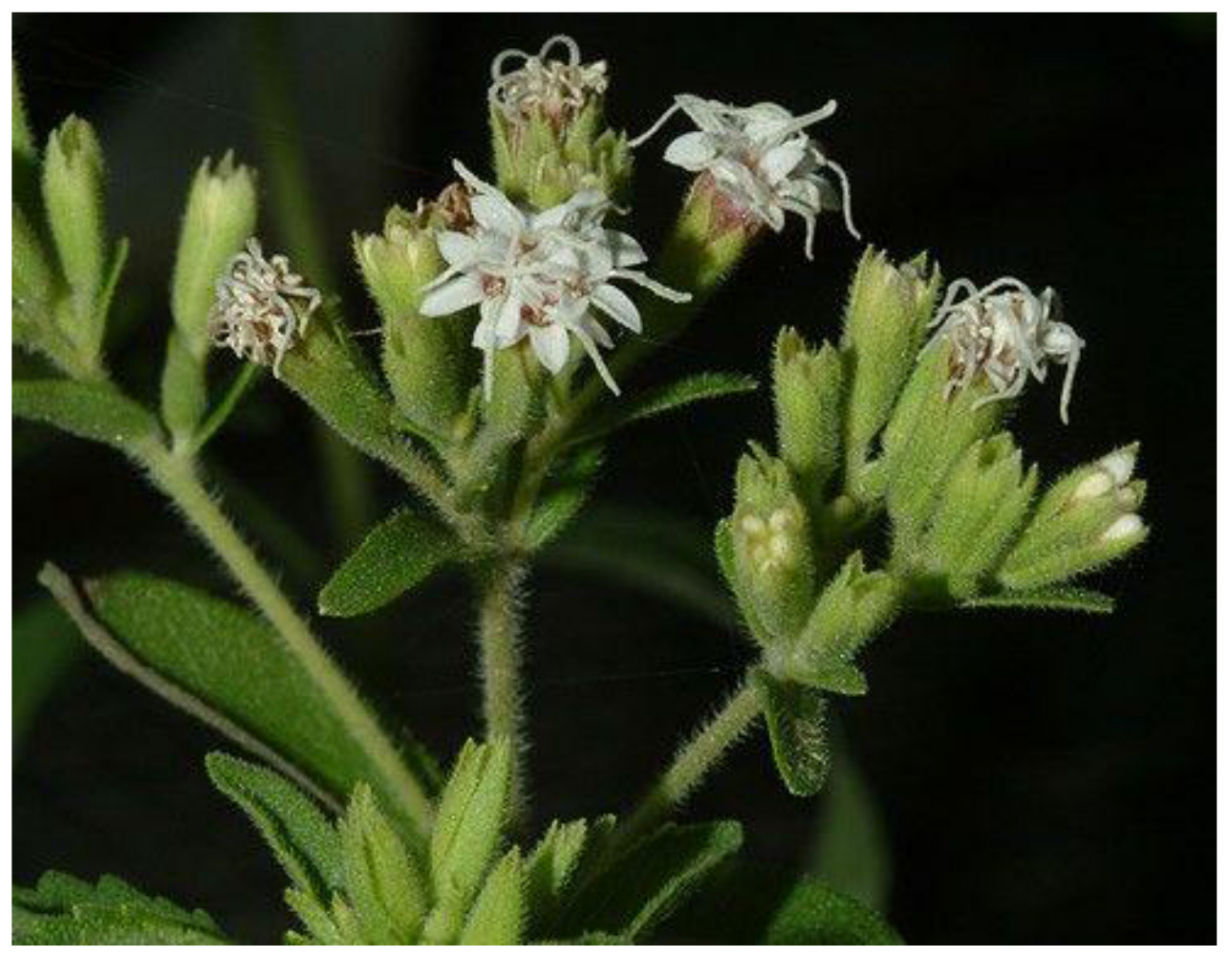
Morphology of *Stevia rebaudiana* Bertoni. The image shows green leaves, branched stems, capitula, and small white flowers, illustrating the typical botanical features of stevia. Photograph © Ori Fragman-Sapir. Reproduced with permission. Available at POWO Science Kew.

*Stevia rebaudiana*, formerly known as *Eupatorium rebaudianum* Bertoni, is a perennial herbaceous plant in the genus Stevia of the family *Asteraceae* (14). Its morphological characteristics are shown in Figure 1, and its taxonomic classification is presented in Table 1. The plant is native to the Amambay Mountains on the border between Paraguay and Brazil, with a natural distribution that includes Paraguay, southeastern Brazil, and west-central Brazil (34). During plant growth, SGs continuously accumulate in the leaves and generally reach their highest levels from the late vegetative stage through the budding stage, then gradually decline after flowering begins. The leaf developmental stage and cultivation conditions can markedly affect SG composition and the bitter and sweet taste profiles of stevia leaves (17). Therefore, the late vegetative to budding stage is generally considered an appropriate harvest period for stevia (35).

**Table 1:** Taxonomic classification of *Stevia rebaudiana* Bertoni.

Taxonomic level	Classification
Kingdom	Plantae
Phylum	Streptophyta
Class	Equisetopsida
Subclass	Magnoliidae
Order	Asterales
Family	Asteraceae
Genus	Stevia
Species	*Stevia rebaudiana*

In traditional use, the local Guarani people referred to stevia as “kaa-hee,” meaning “sweet herb” or “honey leaf” (14). Indigenous communities used stevia as a natural sweetness to improve the flavor of beverages, particularly as a sweetener for maté tea (36). In addition, stevia has been reported in ethnobotanical literature for contraceptive-related and diabetes-related folk uses (33). These traditional practices provide important evidence of stevia's early dietary and medicinal uses.

With the increasing demand for natural sweeteners and the expansion of global cultivation, stevia has gradually developed from a native South American plant into a globally cultivated economic crop, with production now extending across the Americas, Asia, Europe, and other regions (14, 33).

### 4.2 Chemical composition of stevia extracts

**Figure 2:**
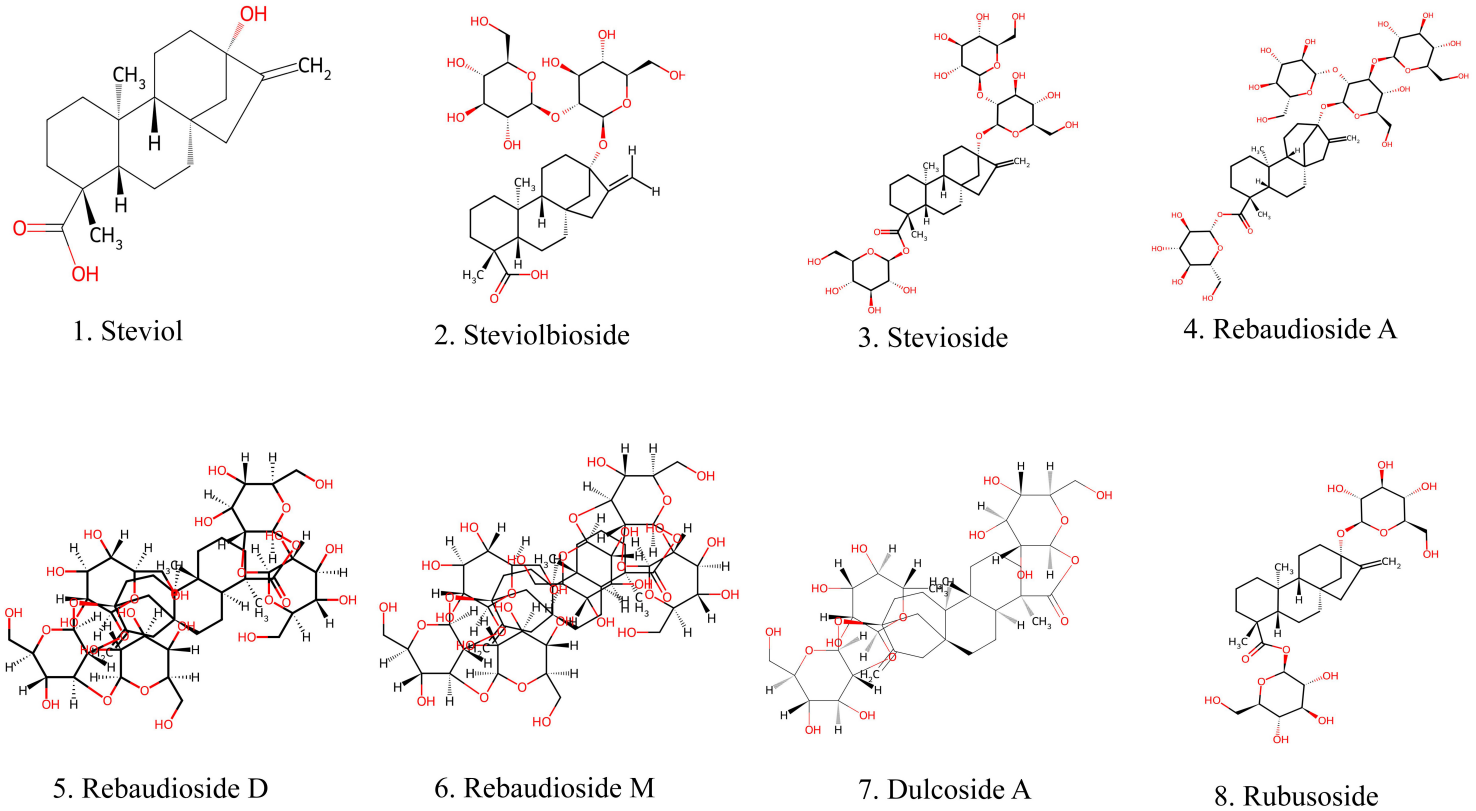
Chemical structures of steviol and representative steviol glycosides. Chemical structures obtained and adapted from ChemSpider, Royal Society of Chemistry with permission: CSID 398979, 10209449, 390625, 5294031, 24606173, 24606174, 24606167, 23334893. Accessed 19 07 2026.

Stevia leaves contain approximately 150 bioactive chemical constituents, mainly including diterpenes and their derivatives, flavonoids, phenylethanoids, phenolic compounds, and volatile oils (14). Among these constituents, SGs are the major sweet-tasting compounds in stevia (17). More than 60 SGs have been identified, and their structural diversity is mainly determined by differences in glycosylation patterns at the C13 and C19 positions of the steviol backbone (13). The chemical structures of representative SGs are shown in Figure 2. In addition to SGs, stevia also contains alkaloids, sterols, polysaccharides, fatty acids, amino acids, and purine compounds (15). Fresh stevia leaves have a high water content of approximately 80%−85% and contain proteins, dietary fiber, monosaccharides, lipids, essential oils, vitamin C, β-carotene, vitamin B2, vitamin B1, and minerals such as cobalt, magnesium, iron, potassium, and phosphorus (37, 38). Together, these constituents provide a chemical basis for the nutritional value, flavor characteristics, and potential bioactivity of stevia, supporting its application in health-oriented low-sugar food formulations (Table 2).

**Table 2:** Major chemical constituents of stevia and their potential food-related value.

Constituent category	Representative compounds	Potential bioactivities and health benefits	Food-related value	Ref.
Diterpenes and derivatives	Reb A, Reb M, Reb C, steviol, STV, and other SGs	Potentially contribute to regulating blood glucose, blood pressure, and body weight.	High sweetness intensity and low energy value; can be used as sucrose substitutes.	(14, 134, 136, 200)
Phenolic compounds and derivatives	Flavonoids, such as quercetin, kaempferol, and their glycosides; phenolic acids, such as chlorogenic acid, isochlorogenic acids, caffeic acid, and ferulic acid.	Antioxidant, anti-inflammatory, antibacterial, digestive-enzyme inhibitory, and potential antidiabetic activities; antitumor evidence remains preliminary	May improve flavor complexity, enhance antioxidant capacity, extend shelf life, and increase nutritional value.	(113, 201–203)
Polysaccharides	Soluble polysaccharides	Immunomodulatory and antioxidant activities, with potential benefits for gut health	Improve texture and viscosity, enhance mouthfeel, and increase dietary-fiber-related nutritional value.	(204, 205)
Volatile oils	Terpenes and aromatic compounds, such as limonene, linalool, and menthol	Antibacterial, antiviral, and anti-inflammatory activities; some compounds may have soothing effects	Improve aroma and flavor and enhance sensory appeal	(15)
Amino acids and derivatives	Glutamic acid, alanine, and others	Involved in protein synthesis, immune function, and metabolic homeostasis	Improve taste, such as by increasing umami and softening sweetness, while contributing to nutritional value	(204)
Fatty acids and derivatives	α-Linolenic acid, oleic acid, and others	Potential cardioprotective, cholesterol-regulating, and metabolism-improving effects	Improve flavor and provide some essential fatty acids	(134, 204)

Reb A, rebaudioside A; Reb C, rebaudioside C; Reb M, rebaudioside M; SGs, steviol glycosides; STV, stevioside; Ref., reference.

This table summarizes the major categories of active constituents in stevia leaves, their representative compounds, and their potential bioactivities and food-related values. In addition to SGs, which provide the primary sweet taste, non-glycoside constituents such as phenolic compounds, polysaccharides, volatile oils, amino acids, and fatty acids may also influence the flavor profile, nutritional properties, and the potential for functional food development of stevia extracts.

Structurally defined and highly purified SGs, such as STV, Reb A, and Reb M, with total SGs content typically not less than 95% on a dry-weight basis, are among the most widely used stevia-derived sweeteners in the food industry (39). In contrast, crude or conventional stevia extracts, including aqueous, ethanolic, and stevia residue extracts, are complex mixtures. In addition to certain amounts of SGs, these extracts contain other compounds, including flavonoids, phenolic acids such as chlorogenic acid and caffeic acid, and essential oils (17). These secondary metabolites may introduce additional bitterness, astringency, or plant-like aftertastes, thereby affecting the overall sensory experience of the final product (17).

## 5 Molecular basis of sweet taste perception and sensory responses

### 5.1 Taste receptor mechanisms of steviol glycosides

Sweet taste perception in humans is primarily mediated by the taste receptor type 1 member 2/type 1 member 3 (T1R2/T1R3) heterodimer (40). This receptor belongs to the class C G protein-coupled receptor (GPCR) family. It is primarily expressed in oral type II taste receptor cells, where it participates in the initial transduction of sweet taste signals to the central nervous system (5). T1R2/T1R3 consists of an extracellular Venus flytrap module (VFTM), cysteine-rich domain (CRD), and seven-transmembrane domain (TMD), enabling the recognition of diverse sweet-tasting molecules, including natural sweeteners, artificial sweeteners, and sweet-tasting peptides, through distinct binding sites (5, 41).

The perception of sweetness by SGs is closely associated with their receptor binding patterns. Studies have shown that stevioside mainly binds to the VFTM of human T1R2, whereas natural sugars, such as glucose and sucrose, as well as sucralose, can bind to the VFTMs of both T1R2 and T1R3 (41). This differential recognition pattern may explain the differences between SGs and natural sugars in sweetness intensity, sweetness onset, and aftertaste characteristics (41).

In addition, the binding mode and binding affinity of SGs with the human sweet taste receptor hT1R2-hT1R3 are closely related to their perceived sweetness. Hydrogen bonding, hydrophobic interactions, and glycosyl structures all affect the stability of glycoside-receptor binding (42). Meanwhile, SGs can mediate the perception of bitterness through interactions with hTAS2R4 and hTAS2R14 receptors (43). Structural features, including glycoside chain length, pyranose substitution, and C16 double bond, jointly influence the sweet and bitter taste profiles of SGs (43). These findings provide a theoretical basis for the rational design of novel glycosides with high sweetness and low bitterness. Therefore, the sensory performance of SGs is not determined solely by sweetness intensity, but is jointly shaped by sweet receptor binding, bitter receptor activation, and the integration of multiple taste signaling pathways (17, 44).

Although different receptor systems mediate sweetness, bitterness, and umami, their downstream signaling pathways share several common features. Receptor activation can initiate the phospholipase C β2 (PLC-β2) signaling pathway, induce inositol 1,4,5-trisphosphate (IP_3_)-mediated intracellular Ca^2+^ release, and activate transient receptor potential cation channel subfamily M member 5 (TRPM5).

This process subsequently promotes calcium homeostasis modulator 1 (CALHM1)-mediated adenosine triphosphate (ATP) release, transmitting taste signals to the central gustatory pathways (45–49).

Overall, the sweet and bitter taste characteristics of SGs are jointly determined by receptor binding patterns, glycosyl substitution structures, and the integration of multiple taste signaling pathways. These mechanisms provide a molecular basis for screening low-bitterness glycosides, optimizing glycoside blending ratios, and developing low-sugar food formulations with sucrose-like sensory profiles.

### 5.2 Extraoral sweet taste perception and metabolic signaling pathways

T1R2/T1R3 receptors are not restricted to oral taste cells but are also distributed in the gastrointestinal tract, pancreatic β cells, and adipose tissue, where they participate in carbohydrate sensing, enteroendocrine regulation, and glucose homeostasis (41). Unlike oral sweet taste perception, extraoral sweet taste perception is not limited to sweet recognition but may also contribute to nutrient sensing and regulation of metabolic signals. Nutritive sugars primarily signal through the GPCR–Gαs–cyclic adenosine monophosphate (cAMP) pathway, whereas low-calorie sweeteners more commonly rely on the GPCR–Gβγ-PLCβ2–IP_3_-Ca^2+^-TRPM5 pathway (5). Intestinal nutrient-sensing cells can discriminate between sugars and NNSs. Studies have shown that cholecystokinin (CCK)-labeled neuropod cells in the duodenum can rapidly discriminate nutritive sugars from NNSs and transmit signals to the central nervous system via the vagus nerve (50).

TRPM5 is a key ion channel linking sweet taste perception to metabolic responses (51). Studies have shown that steviol and certain SGs, such as stevioside and Reb A, can enhance TRPM5 channel activity in taste and pancreatic β cells, regulate glucose-induced Ca^2+^ oscillations, promote glucose-dependent insulin secretion, and increase the intensity and duration of sweet taste perception (49). In addition, related studies have reported improvements in hyperglycemia in animal models (12). However, this evidence is mainly derived from animal- and cell-based experiments and cannot be directly extrapolated to glucose-lowering effects in humans.

Accordingly, the relevance of SGs in reduced-sugar foods may extend beyond sweetness replacement and energy reduction to include extraoral nutrient sensing and gut-brain signaling pathways. Nevertheless, formulation design should continue to prioritize proven food technology outcomes, such as sweetness quality, matrix compatibility, and sugar-load reduction, while treating metabolic signaling hypotheses as emerging evidence requiring further human validation.

## 6 Sensory characteristics and optimization strategies of stevia

The effectiveness of stevia as a sugar substitute in sugar-reduced foods depends not only on sweetness intensity but also on sweetness quality, time-intensity profile, aftertaste, off-flavors, oral fullness, and overall flavor harmony (52, 53). Compared with sucrose, stevia has the advantages of high sweetness intensity and low energy value; however, it is still associated with differences in sweetness onset, lingering sweetness, bitterness, herbal notes, metallic sensations, and insufficient mouthfeel (49, 54, 55). These sensory limitations are closely related to the molecular structures of SGs, composition of food matrices, and recognition characteristics of sweet and bitter taste receptors, and they remain important factors limiting the high-level replacement of sucrose by stevia in sugar-reduced foods (53, 55–57).

Table 3 summarizes the major sensory differences between stevia extracts and sucrose in terms of sweetness intensity, sweetness time-intensity profile, aftertaste, off-flavors, mouthfeel, and metabolism-related characteristics. These comparisons may provide a reference for sugar-reduced formulation design, flavor modification, and sensory quality optimization using stevia.

**Table 3:** Comparison of the major characteristics of sucrose and stevia extracts.

Sensory dimension	Sucrose	Stevia extracts/SGs	Common defects or manifestations	Ref.
Sweetness intensity	Serves as the reference sweetener, with a clear and stable sweetness profile	Approximately 50–350 times sweeter than sucrose, depending on the glycoside type and food matrix	High sweetness intensity does not necessarily produce a sugar-like taste; excessive sweetness may amplify aftertaste defects.	(62, 87, 88)
Sweetness onset	Natural and smooth onset of sweetness	The temporal sweetness profile often differs from that of sucrose; some systems show a slower onset or a different peak-sweetness distribution.	Does not fully reproduce the rapid and smooth sweetness onset of sucrose	(60, 90, 206)
Sweetness lingering	Relatively clean finish with limited lingering sweetness	Longer-lasting sweetness, particularly for Reb A; Reb D and Reb M show improved temporal profiles but may still linger longer than sucrose	Lingering sweetness is one of the most common sensory differences between stevia and sucrose	(60, 90, 207)
Bitterness	Almost no obvious bitterness	Bitterness is a typical sensory limitation of stevia, especially in crude extracts, stevioside, and formulations with higher levels of Reb A	Bitterness is often more evident after swallowing	(60, 88, 90, 207)
Bitter aftertaste	Very low bitter aftertaste	Bitter aftertaste is more pronounced than in sucrose; it is common with Reb A and relatively weaker with Reb D and Reb M.	A key factor limiting high-level sucrose replacement with stevia	(60, 90, 207)
Licorice-like/herbal notes	Generally absent	Licorice-like, herbal, or plant-like notes are common, especially in beverage systems.	Consumers may describe these notes as medicinal or plant-like	(88, 206, 207)
Metallic/artificial perception	Generally absent	Metallic or artificial sweet sensations may occur, particularly at high doses or when stevia is used alone.	More noticeable at high doses or when stevia is used alone	(60, 207)
Astringency	Usually low	Astringency or puckering sensations may increase in some juice- and tea-based systems.	Strong interactions may occur with acidity, tea polyphenols, and fruit-flavor matrices.	(90, 206)
Cleanliness of sweetness profile	High; closely matches consumer expectations of standard sweetness	Usually lower than that of sucrose; Reb M and Reb D generally show cleaner profiles than Reb A and stevioside	Blending, masking, or flavor modulation is often needed to improve acceptability	(60, 88)
Oral body and fullness	Provides sweetness as well as bulk, viscosity, and roundness	As high-intensity sweeteners, stevia compounds lack the bulk and oral fullness provided by sucrose	When used as the sole sucrose substitute, products may taste thin, hollow, or sharp	(87, 206)
Caloric and glycemic effects	Provides energy and increases dietary sugar load	Provides almost no energy and does not directly raise blood glucose	Suitable for sugar-reduction and glycemic-control applications, although health value depends on the overall formulation	(87, 88)

SGs, steviol glycosides; Reb A, rebaudioside A; Reb D, rebaudioside D; Reb M, rebaudioside M; Ref., reference.

This table summarizes the main differences between sucrose and stevia extracts/SGs in sweetness intensity, temporal sweetness characteristics, aftertaste, off-flavors, oral fullness, and caloric and glycemic effects.

### 6.1 Sensory differences among steviol glycosides and their formulation implications

The sweetness of stevia extracts is mainly derived from SGs (17), among which STV and Reb A are the most representative glycosides (15). These compounds share a common steviol aglycone core, namely ent-13-hydroxykaur-16-en-19-oic acid; however, differences in their glycosylation patterns lead to substantial variations in sweetness intensity, bitterness, aftertaste, and overall flavor profile (17, 44, 49). Table 4 summarizes the sensory properties, structural features, and formulation relevance of the major SGs.

**Table 4:** Sensory characteristics, structural features, and formulation significance of major SGs.

Glycoside	Natural abundance	Relative sweetness vs. sucrose	Main sensory profile	Key structural feature	Sweetness perception-related feature	Main limitation	Formulation relevance	Ref.
STV	One of the major glycosides; approximately 22%−62%	250–300×	High sweetness, but with pronounced bitterness, licorice-like aftertaste, and lingering sweetness	Basic glycosylation pattern; fewer glucose moieties than Reb A	Relatively strong interaction with sweet taste receptors and high sweetness potency	Pronounced aftertaste and relatively poor clean sweetness profile	Cost-effective; suitable for partial sugar replacement or use in blends with bulking agents and flavor modulators	(17, 42)
Reb A	One of the major glycosides; approximately 5%−22%	250–450×	Cleaner sweetness than STV and lower bitterness, but lingering sweetness and residual off-notes may remain at high doses.	More highly glycosylated structure; one additional glucose moiety at C13 compared with STV	Strong binding to sweet taste receptors and high sweetness potency	Residual bitterness and aftertaste may still occur at higher doses	One of the most widely commercialized SGs and a benchmark ingredient in reduced-sugar beverages and foods	(17, 42)
Reb C	Low abundance; approximately 1%−2% in dried leaves	20–30×	Lower sweetness intensity and may contribute mild astringency or flavor complexity.	Contains rhamnose and has a glycosylation pattern distinct from Reb A	Lower receptor-binding capacity than Reb A	Insufficient sweetness for stand-alone use	More suitable as a flavor-balancing component than as a primary sweetener	(17, 42)
Reb D	Very low natural abundance; minor glycoside	200–220×	Lower bitterness, shorter aftertaste, and a more sucrose-like profile	Additional glucose moiety at C19 and a more favorable glycosylation pattern for taste quality	Better sweetness perception than Reb A and STV	Low natural abundance and higher production cost	High-value ingredient for premium reduced-sugar formulations and taste-quality improvement	(17, 44)
Reb M	Extremely low natural abundance; often < 0.5% in the plant	200–350×	Smooth sweetness, fewer bitter or off-notes, and a more sucrose-like profile	Extended glycosylation pattern with additional glucose moieties at C19	Generally shows one of the most favorable sweetness perception profiles	Very low natural abundance; production depends on enzymatic conversion or biomanufacturing	Next-generation SG for high-quality beverages and reduced-sugar products	(17, 61, 62)
Dulcoside A	Low abundance	~30×	Lower sweetness and more noticeable bitterness	Contains rhamnose and is structurally related to some minor glycosides	Moderate sweet taste receptor interaction	Limited sweetness potency and sensory quality	May be used as a flavor modifier or auxiliary formulation component	(17, 42)
Other minor glycosides, such as Reb B, Reb E, Reb F, and rubusoside	Extremely low natural abundance	Usually < 50×	Low sweetness intensity and complex flavor profiles	Diverse minor glycosylation patterns	Generally low sweet taste receptor-binding capacity	Not suitable as primary sweeteners	Useful for flavor balancing or structure–flavor relationship studies	(17, 42)

Reb A, rebaudioside A; Reb B, rebaudioside B; Reb C, rebaudioside C; Reb D, rebaudioside D; Reb E, rebaudioside E; Reb F, rebaudioside F; Reb M, rebaudioside M; SGs, steviol glycosides; STV, stevioside; Ref., reference.

Major SGs, such as STV and Reb A, are commonly associated with pronounced bitterness, licorice-like aftertaste, and prolonged sweetness lingering (17, 44, 49). In contrast, minor SGs, particularly rebaudioside D (Reb D) and rebaudioside M (Reb M), generally show a faster onset of sweetness, lower bitterness, shorter aftertaste, and a more sucrose-like flavor profile (49, 58–60). Among them, Reb M is characterized by a smoother sweetness profile and fewer off-flavors, and is perceived by consumers as closer to sucrose (59). It is therefore widely regarded as one of the next-generation SGs with considerable potential for further development (61, 62). The low natural abundance of Reb D and Reb M in stevia leaves has promoted the development of production technologies such as enzymatic conversion, microbial fermentation, and biocatalysis (58, 59, 61). For example, an engineered *Saccharomyces cerevisiae* biocatalytic system enabled the efficient conversion of STV to Reb M, yielding 12.5 g/L and achieving a conversion rate of 77.9%, providing a feasible strategy for industrial production (63).

Minor SGs identified in recent years, including rebaudioside D17 (Reb D17), rebaudioside D2 (Reb D2), rebaudioside T (Reb T), rebaudioside U (Reb U), rebaudioside U3 (Reb U3), rebaudioside E19 (Reb E19), and rebaudioside S (Reb S), also exhibited distinct glycosylation patterns. Among these compounds, Reb D17 and Reb U3 contain β-1 → 4 glycosidic linkages at the C19 position, whereas Reb E19 may serve as a potential biosynthetic intermediate (17, 56, 64–66). Future studies should clarify the sensory characteristics, structure–flavor relationships, and food application potential of these recently identified glycosides.

### 6.2 Improvement of sensory quality through glycoside blending and structural modification

The core objective of sensory optimization for stevia is not merely to match the sweetness intensity of sucrose but also to reduce bitterness, astringency, a licorice-like aftertaste, and lingering sweetness, while improving sweetness clarity, clean flavor, and overall acceptability (16, 17).

Glycoside blending is an important strategy for improving the sensory quality of stevia. Blending Reb A with Reb M can significantly improve the clean sweetness and overall acceptability (60). The combination of glycosylated rebaudioside A (G-Reb A) with maltitol can reduce bitterness and astringency, suggesting that glycosylation and sugar alcohol blending may be an effective approach to improving the sensory quality of SGs (67). Recent studies have reported five minor SGs containing rhamnose or xylose residues, among which rebaudioside FX1 shows a well-balanced sweetness profile and low bitterness, indicating its potential for application in low-sugar foods (68). Consumer sensory evaluations further confirmed that specific glycoside combinations and blending ratios can significantly enhance sweetness, purity, and overall acceptability (16). However, most of these findings are based on aqueous solutions or simple model systems and require further validation in real food matrices.

Glycosylation-based structural modification represents another key strategy. Molecular simulations and structural modification studies have shown that adjusting glycosylation at the C19 position can reduce bitter receptor binding and enhance sweet receptor binding, thereby reducing bitterness while maintaining sweetness (69). Dynamic sensory studies further indicated that increasing the number of glucose moieties at the C13 position can enhance sweetness intensity and the rate of sweetness onset, whereas increasing glycosylation at the C19 position may delay bitterness onset, reduce bitterness intensity, and accelerate aftertaste dissipation (55). These findings suggest that the position of glycosyl substitution and the length of glycosyl chains are important structural determinants of sweetness quality and aftertaste performance in SGs.

From a production perspective, enzymatic conversion and microbial fermentation offer feasible routes to obtain SGs with improved sensory quality. The key enzyme UDP-glucosyltransferase 76G1 (UGT76G1) can catalyze the β(1 → 3)-glucosylation conversion of Reb D into Reb M and other high-quality glycosides, providing a theoretical and technical basis for increasing the yield of desirable SGs (58, 70). Because Reb D and Reb M occur at low natural abundance in stevia leaves, such biomanufacturing technologies are important for improving the industrial application value of SGs.

In addition, aftertaste formation may be related not only to receptor recognition but also to oral adsorption/desorption behavior and receptor-binding kinetics (55). This suggests that future sensory optimization should not focus solely on the sweetness intensity of individual glycosides but should integrate oral retention behavior, food matrix interactions, and dynamic sensory changes into the formulation design.

Taken together, these approaches suggest that glycosylation modification, enrichment of sensory-favorable glycosides, and blending with sugar alcohols or rare sugars are practical routes for reducing bitterness, astringency, and lingering sweetness in low-sugar beverages, dairy products, confectionery products, and bakery products.

### 6.3 Technological functions of sucrose and formulation challenges during sugar reduction

In reduced-sugar food formulation, sucrose reduction is not merely a matter of replacing sweetness. Beyond sweetness, sucrose contributes to volume development, texture and mouthfeel formation, viscosity control, regulation of water activity and freezing point, browning reactions, fermentation behavior, crystallization processes, and shelf-life stability (20, 21, 71, 72). Therefore, the development of reduced-sugar foods requires targeted formulation redesign to address quality defects arising from sucrose reduction across different food matrices.

In bakery products, sucrose reduction may alter batter rheology, starch gelatinization, protein denaturation, baking-related phase transitions, water distribution, browning reactions, and the formation of aerated structures, thereby affecting product volume, texture, color, shelf-life stability, and the crumb structure of cakes or bread (72, 73). In beverages and dairy products, sugar reduction may weaken rounded sweetness, oral fullness, viscosity, aroma perception, and flavor harmony, while making the bitterness, metallic notes, or lingering aftertaste of high-intensity sweeteners more perceptible (74, 75).

In hard confectionery and chocolate, sucrose reduction may affect water activity, crystallization behavior, rheological properties, hardness, chewiness, color, melting characteristics, and storage stability (20, 76–78). In gelled foods such as gummy candies, jams, and jellies, sucrose reduction may affect water activity, total soluble solids (TSS), crystallization behavior, sol-gel transition, and the stability of hydrocolloid networks, thereby altering product hardness, gumminess, syneresis stability, color, mouthfeel, and storage quality (76, 79). Among these products, gummy formulations generally require adjustments to the sucrose-to-glucose syrup ratio and gelling components, such as gelatin and starch (79). In contrast, jams and jellies rely more on hydrocolloids, sugar alcohols, dietary fibers, acidity regulators, and optimization of processing parameters to maintain gel structure and sensory quality (76).

In ice cream and frozen desserts, sucrose reduction affects ice formation, freezing-point control, serum-phase viscosity, hardness, and melting behavior. Therefore, sugar replacement should consider not only sweetness but also solute molecular weight, ice content, unfrozen-phase properties, and solids balance (21, 80, 81). In addition, in fermented foods, reducing or replacing sucrose, which serves as a fermentable substrate, may affect microbial growth, acidification kinetics, the formation of flavor compounds, and product stability (82–84).

Given these quality defects, sucrose reduction should be approached through matrix-specific formulation redesign. One category of strategies aims to reduce sucrose addition while enhancing the perceived sweetness efficiency of the remaining sucrose within the food matrix, through stepwise sugar reduction, formulation modification, multisensory enhancement, sugar particle-size control, and optimization of spatial sweetness distribution (20, 76). Another category involves replacing part of the sucrose with high-intensity sweeteners; however, because these sweeteners are used at low levels, additional formulation components are often required to compensate for the non-sweet technological functions lost after sucrose reduction (72, 85, 86).

### 6.4 Food formulation strategies involving stevia

Stevia-derived sweeteners have been used in a wide variety of reduced-sugar products, including cookies, muffins, cakes, chocolate, desserts, ice cream, and jams (25). Because SGs are used at low levels, they are commonly combined with sugar alcohols, rare sugars, dietary fibers, or other bulking and texture-modifying systems to compensate for the loss of solids, bulk, oral fullness, texture, and sucrose-like temporal sweetness profiles after sucrose reduction (17, 62, 76, 87). In practical product development, SGs are commonly combined with erythritol, xylitol, D-allulose, dietary fiber, or other matrix components. Such combinations can compensate for the loss of bulk, viscosity, oral fullness, and texture after sucrose reduction (88, 89).

Evidence from food applications indicates that these SG-based combinations can partly mask the bitter aftertaste, reduce bitterness peaks, enhance sweetness clarity, and improve mouthfeel, thereby increasing the formulation value of SGs in reduced-sugar products (90–93).

In low-calorie apricot nectar, Reale et al. (94) compared a sugar-free formulation, a 10% sucrose formulation, and formulations containing 0.07 or 0.14% stevia. Stevia-sweetened nectars reduced the energy value from 86 kcal in the 10% sucrose control to 49 kcal, and the 0.07% stevia formulation showed consumer acceptability comparable to that of the sucrose-containing formulation. In gummy products, replacing up to 60% of sugar with stevia maintained acceptable texture and children's acceptance levels comparable to the full-sugar formulation, supporting the feasibility of partial sugar replacement in gelled confectionery (95). In chocolate milk, partial replacement of sucrose with sweetener systems containing stevia or monk fruit at a 75:25 sucrose/alternative sweetener ratio produced sensory profiles close to the sucrose-sweetened control, although bitterness, metallic notes, or aftertaste may still occur (74, 78). In sucrose-free probiotic frozen yogurt, Atallah et al. (75) identified 0.06% stevia, 0.03% sucralose, and 3% sorbitol as the optimal levels based on preliminary sensory and texture analyses; maltodextrin and polydextrose were each used at 7.5% to compensate for the loss of total solids after sucrose removal.

Although sugar alcohols are useful for restoring bulk and improving the sensory profile of stevia-containing formulations, their doses should be carefully controlled because high intake may induce gastrointestinal intolerance, particularly in sensitive consumers, such as individuals with irritable bowel syndrome, people sensitive to fermentable oligosaccharides, disaccharides, monosaccharides, and polyols, and children (89). Therefore, reduced-sugar formulation design requires a balance among sweetness optimization, texture reconstruction, energy control, and gastrointestinal tolerance. Incorporating dietary fiber may provide an additional strategy to improve texture and nutritional quality while potentially supporting gastrointestinal health in sugar-reduced formulations (96).

Food matrix optimization can further improve the performance of stevia in reduced-sugar products. In low-sugar yogurt, protein beverages, functional gummies, reduced-sugar rice crackers, and reduced-sugar muffins, SG blending combined with the incorporation of dietary fiber or protein-based matrix components and optimization of processing parameters can improve sweetness clarity, smoothness, bulk, and texture, while enhancing functional properties (57, 91, 92, 97, 98). In fermented foods and functional beverages, stevia may affect microbial activity and modulate the formation of flavor compounds and polyphenols (99, 100). In addition, barley nuruk-fermented stevia has been reported to exhibit antioxidant, α-glucosidase inhibitory, and anti-inflammatory activities, suggesting its potential as a functional food ingredient (101). However, quantitative data on the effects of strain combinations and fermentation conditions on SG stability and flavor generation remain limited.

Overall, the effective application of stevia in reduced-sugar products lies in synergistic formulation design rather than simple sweetness replacement (87, 102). Future product development should integrate SG selection, sweetener blending, matrix reconstruction, processing optimization, gastrointestinal tolerance, and consumer sensory evaluation to improve the sweetness quality, nutritional value, and product stability.

## 7 Central neural basis of stevia-related sensory limitations and optimization strategies

Although SGs can be matched with sucrose in terms of perceived sweetness, they may elicit weaker activation than sucrose in brain regions associated with taste processing, palatability, and reward, including the insula, orbitofrontal cortex (OFC), and striatum (44, 103, 104). At high concentrations, stevia is also associated with sweetness lingering, enhanced herbal aroma, prolonged bitterness, and insufficient oral fullness (44, 52).

Animal studies have shown that Reb A intake can alter activation patterns in brain regions related to the mesolimbic dopaminergic reward system (105). Human studies further indicate that even when sucrose and stevia are similar in perceived sweetness, they elicit significantly different neural processing times for sweet taste signals, approximately 150.9 ms and 203.8 ms, respectively (106). These studies indicate that the sensory limitations of stevia may arise not only from peripheral receptor recognition but also from differences in central neural processing (52). However, the available neuroimaging evidence remains limited and should be interpreted cautiously.

Reb D-based blending strategies provide a potential approach for improving the sucrose-like sensory experience of SGs. Functional magnetic resonance imaging (fMRI) studies have shown that the synergistic incorporation of glucose and sodium ions into a Reb D system may partially mimic the activation characteristics of the T1R2/T1R3 sweet taste receptor and sodium-glucose cotransporter 1 (SGLT1) pathways, making subjective palatability and OFC neural responses more similar to those induced by sucrose (44). Recent evidence from gut–brain signaling provides a new perspective for explaining this difference: nutritive sweeteners and NNSs may engage distinct receptor and neurotransmitter pathways in the duodenum, thereby generating different vagal signals to the central reward system (50).

When stevia is combined with a flavor modifier (FMP), additive activation can occur in the postcentral gyrus, parietal cortex, and occipital cortex. Meanwhile, responses in brain regions associated with pleasure and oral fullness, such as the hypothalamus and nucleus accumbens, are enhanced, whereas amygdala responses to bitterness are suppressed (52, 107). These findings suggest that formulation strategies may improve the sensory experience of stevia to some extent. However, their practical effectiveness is likely to depend on sweetener composition, food matrix characteristics, exposure conditions, and consumer sensory heterogeneity.

Overall, current evidence indicates that specific blending strategies can partially approximate the sucrose-like sensory experience of SGs, but they still cannot fully reproduce the physiological and neural reward effects of sucrose (44, 50, 52). Future studies should combine multimodal neuroimaging, dynamic sensory evaluation, real food matrices, repeated-exposure designs, and consumer acceptance testing to establish more reliable sensory optimization strategies for stevia-containing reduced-sugar foods.

## 8 Nutritional and metabolic effects of stconstituents

### 8.1 Glycemic homeostasis and insulin sensitivity

When SGs are incorporated into reduced-sugar formulations as sucrose replacers, their most direct nutritional contribution is the reduction in added sugar content, energy density, and potential glycemic load. In addition to this substitution effect, preclinical studies have reported several glucose-regulatory actions of stevia-derived constituents. Regarding insulin secretion and glucose handling, several stevia-derived constituents have been reported in insulinoma cell models (INS-1 cells) to modulate glucose transporter type 4 (GLUT4)-related glucose uptake, and promote insulin release (37, 108–111). For peripheral glucose handling, steviol and stevioside have been associated with PI3K/GLUT4-related signaling and enhanced glucose uptake (108, 112). SGs may also enhance TRPM5 activity in β cells, thereby supporting glucose-dependent insulin secretion and linking sweet-taste signaling with metabolic responses (49). In hepatic glucose metabolism, stevia-derived polyphenolic compounds have been reported to increase glucokinase activity, promote hepatic glucose utilization, and suppress hepatic glucose output, which may contribute to improved glycemic control and reduced hyperglycemia-related oxidative injury in experimental models (113). Animal studies have further indicated that Reb M has more favorable metabolic effects than fructose, including improved glycemic indicators, enhanced insulin sensitivity, and reduced body weight gain in obese mice (12, 114).

The nutritional evidence summarized in Table 5 suggests that, in human studies, stevia-derived ingredients and sweeteners may provide certain glycemic benefits in some study settings. For example, a preliminary human study showed that a blended sugar substitute containing erythritol, xylitol, and stevia elicited a lower glycemic response than a glucose reference (115).

**Table 5:** Narrative stratification of nutritional evidence for stevia-derived ingredients and sweeteners.

Evidence level	Main nutritional effects	Representative references
Stronger human evidence	Sugar and energy intake reduction in food and drink	(118, 208, 209)
Moderate human evidence	Lower postprandial glycemic response when stevia-derived sweeteners replace sucrose in specific food matrices	(115, 125, 210, 211)
	Favorable modulation of postprandial glucose–insulin responses	(212–214)
Limited human evidence	Body weight regulation via energy intake reduction	(124, 125, 215)
	Benefits related to cardiometabolic health	(6, 162, 216)
	Reduced appetite	(217, 218)
	Oral health including low cariogenic potential	(153, 219)
	Increased subjective satiety	(220, 221)
	Blood pressure regulation	(137, 222, 223)
	Generally no significant effects on gut microbiota	(162, 224)
Mainly preclinical evidence	Metabolic regulation (glucose–insulin pathways)	(108–110, 114).
	Modulation of lipid metabolism	(128, 131)
	Antioxidant activity	(141, 160)
	Anti-inflammatory activity	(143, 144, 225)
	Modulation of gut barrier function	(160, 175)

This table provides a narrative stratification of the nutritional evidence for stevia-derived ingredients and sweeteners, aiming to summarize the current evidence landscape and distinguish relatively well-supported nutritional effects from hypothetical mechanistic effects and emerging research directions requiring further validation.

However, most of the current mechanistic evidence comes from *in vitro* and animal studies. Short study durations, heterogeneous formulations, and differences in comparator sweeteners, food matrices, and study populations often limit the available human studies. Therefore, the primary value of SGs in reduced-sugar foods remains their ability to reduce sugar load and optimize the nutritional structure of formulations. In contrast, their specific metabolic regulatory mechanisms and long-term effects in humans require further investigation (12).

### 8.2 Body weight management and energy intake

In 2022, more than 390 million children and adolescents aged 5–19 years were overweight worldwide, among whom approximately 160 million were living with obesity; in adults, approximately 2.5 billion were overweight, and 890 million were affected by obesity (116, 117). Excessive intake of free sugars is associated with poor dietary quality, unhealthy weight gain, obesity, non-communicable diseases, and dental caries (118). Therefore, reducing consumption of sugar-sweetened beverages and high-sugar foods remains an important dietary strategy for body-weight management (119).

Current evidence suggests that replacing sucrose with LNCSs may produce modest improvements in metabolic indicators, including body weight, BMI, body fat percentage, fat mass, waist circumference, and intrahepatic lipid content (120–122). Compared with commonly used artificial NNSs, SGs are barely absorbed or metabolized in their intact form in the human body. Instead, they are hydrolyzed by gut microbiota in the colon to steviol, which is subsequently absorbed, glucuronidated in the liver, and excreted (87). SGs can be used as sucrose substitutes in various low-sugar foods, helping to reduce energy density and added sugar content (13). Related studies suggest that SGs exert minimal biological effects on hypothalamic cells and are less likely to induce obesity-related central leptin resistance (123). A large long-term study involving 1,893 children and adolescents aged 6–15 years found no increase in body fat content associated with regular SG intake, suggesting that stevia has potential as a sugar substitute in the pediatric population (124). In adults, a 12-week randomized open-label trial showed that stevia consumption did not significantly affect glucose or insulin responses, and was associated with slightly improved weight maintenance and reduced energy intake (125).

However, according to the 2023 WHO guidelines on NSSs, NSSs, including stevia and its derivatives, should not be regarded as a stand-alone long-term strategy for body-weight control or chronic disease prevention (126). The conclusions of the systematic review underpinning this guideline suggest that long-term NSS use may be associated with increased risks of type 2 diabetes, cardiovascular diseases, and adult mortality.

Overall, SGs may have supportive value in body weight management. However, these findings should not be interpreted as evidence that NSSs are, by themselves, an effective long-term strategy for body weight control (126). Their role should be considered within the broader context of dietary patterns, the overall reduction of free sugars, energy balance, and food matrix reformulation.

### 8.3 Lipid metabolism and cardiometabolic risk

Dysregulated lipid metabolism is closely associated with obesity, diabetes, non-alcoholic fatty liver disease (NAFLD), and cardiovascular diseases (CVD) (127). In the context of low-sugar food development, stevia may be relevant to cardiometabolic health through both nutritional substitution and bioactive modulation. As a sucrose substitute, it can reduce energy intake and dietary sugar load, whereas certain stevia-derived constituents may influence lipid metabolism, oxidative stress, and low-grade inflammation.

In terms of lipid metabolism, SGs are among the most extensively studied bioactive constituents. *In vitro* and animal studies have shown that SGs can enhance the phosphorylation of AMP-activated protein kinase (AMPK) and acetyl-CoA carboxylase (ACC), inhibit the expression of peroxisome proliferator-activated receptor γ (PPARγ), CCAAT/enhancer-binding protein α (C/EBPα), sterol regulatory element-binding protein 1c (SREBP-1c), and fatty acid synthase (FAS), promote fatty acid oxidation, and improve mitochondrial function, thereby alleviating insulin resistance-related lipid metabolic disorders (128). Aqueous stevia extract has also been reported to improve lipid metabolism by promoting bile acid excretion, reducing intestinal reabsorption of bile acids, and inhibiting lipid absorption, thereby reducing serum cholesterol and triglyceride levels (129). In addition, stevioside and rebaudioside A may regulate cholesterol metabolism by modulating 3-hydroxy-3-methylglutaryl-coenzyme A reductase (HMG-CoA reductase) activity and low-density lipoprotein receptor (LDLR) expression, suggesting a potential role in cholesterol synthesis regulation and cardiometabolic protection (130). In STZ-induced diabetic rats, stevia extract significantly reduced serum triglyceride levels, and this hypotriglyceridemic effect has been suggested to be potentially associated with PPARα-related regulation of lipid metabolism, including regulation of lipoprotein lipase (LPL), apolipoprotein C-II (apo C-II), hepatic fatty acid uptake, esterification, and mitochondrial fatty acid oxidation (131). These mechanisms suggest that Stevia-derived constituents may improve lipid profiles and reduce hepatic lipid accumulation.

With regard to blood pressure regulation, stevioside, Reb A, and stevia protein hydrolysates have shown certain antihypertensive potential in animal models, possibly through vasodilation, renal diuretic effects, and angiotensin-converting enzyme (ACE) inhibition (132–136). A randomized controlled trial reported that oral stevioside reduced blood pressure in individuals with hypertension (137).

Overall, these findings suggest that stevia may serve as a supportive dietary component of low-sugar dietary patterns aimed at improving cardiometabolic health. However, its long-term cardiovascular benefits require further validation in high-quality human intervention studies.

### 8.4 Antioxidant activity and modulation of low-grade inflammation

*Stevia rebaudiana* Bertoni contains various bioactive constituents, among which polyphenols, flavonoids, and phenolic acids constitute an important chemical basis for the antioxidant activity of stevia (37). These compounds can scavenge free radicals, reduce reactive oxygen species (ROS) levels, and inhibit lipid peroxidation, thereby alleviating oxidative stress-related cellular damage (37, 138–140).

*In vitro* and animal studies have shown that stevia extracts or phenolic-rich fractions can increase the activities of antioxidant enzymes, including superoxide dismutase (SOD), catalase (CAT), and glutathione peroxidase (GPx), while reducing the formation of lipid peroxidation products such as malondialdehyde (MDA) (138, 140, 141). For example, in HepG2 cell models, a stevia ethanolic extract reduced H_2_O_2_-induced intracellular ROS levels and increased glutathione (GSH) content, suggesting potential cellular antioxidant protection (139). In addition, 2,2-diphenyl-1-picrylhydrazyl (DPPH), 2,2′-azino-bis (3-ethylbenzothiazoline-6-sulfonic acid) (ABTS^+^), and hydroxyl radical (·OH) scavenging assays showed that the ethyl acetate extract (EAE) of stevia exhibited strong free radical scavenging capacity and could attenuate oxidative DNA damage (138, 142). Papaefthimiou et al. further supported the antioxidant activity of stevia leaf extracts through a systematic review and meta-analysis (141).

Regarding the modulation of low-grade inflammation, stevioside has been shown to downregulate tumor necrosis factor-α (TNF-α), interleukin-1β (IL-1β), interleukin-6 (IL-6), and nitric oxide (NO) levels in THP-1 cells and animal inflammatory models, while inhibiting the activation of the inhibitor of the nuclear factor kappa-B kinase β/nuclear factor kappa-B (IKKβ/NF-κB) signaling pathway (143–145). The regulation of oxidative stress and inflammatory responses may partially mediate these effects. Notably, in the absence of lipopolysaccharide (LPS) stimulation, stevioside itself can also induce the release of TNF-α, IL-1β, and NO from THP-1 cells (144), suggesting that its immunomodulatory effects may depend on the dose, cellular status, and stimulation conditions. In addition, the antioxidant and anti-inflammatory activities of stevia-related constituents may help alleviate inflammation-related tissue injury. Still, their actual anti-inflammatory and tissue-protective effects require further validation in human studies (113).

Overall, the antioxidant and low-grade inflammation-modulating effects of stevia-derived constituents may provide a potential basis for the functional development of low-sugar foods, particularly products designed for oxidative stress management, metabolic health, and natural plant-based formulations.

### 8.5 Oral health and dental caries risk

Sucrose is an important dietary factor in the development of dental caries. It can be fermented by oral bacteria, producing organic acids that lower oral pH and promote the demineralization of enamel and dentin, thereby increasing the risk of caries (146, 147). The WHO guideline indicates that the risk of dental caries increases significantly when free sugar intake exceeds 10% of total energy intake (4).

The low-cariogenic potential of stevia is mainly reflected in three aspects: low acidogenicity, inhibition of cariogenic-associated bacteria, and interference with dental plaque biofilm formation. Studies have shown that leaves or extracts of *Stevia rebaudiana* Bertoni can, to some extent, inhibit the growth of oral cariogenic bacteria and reduce acid production and plaque formation, thereby potentially lowering the risk of sucrose-related caries (37, 148–150). Its potential antimicrobial activity may be associated with the phenolic compounds, polyphenols, terpenes, essential oils, peptides, and alkaloids present in stevia. Among these constituents, phenolic compounds are relatively water soluble and may provide a key chemical basis for the antimicrobial activity of aqueous stevia extracts (151, 152).

Stevia extracts may inhibit the growth of certain oral microorganisms by affecting the bacterial cell wall and membrane permeability and disrupting cellular structural integrity (37, 149, 152). *In vitro* studies further support stevia's low cariogenic potential. Escobar et al. (148) found that stevia reduced biofilm formation and acid production by *Streptococcus mutans* (*S. mutans*), with a minimum inhibitory concentration (MIC) of 25 mg/ml and a minimum biofilm inhibitory concentration (MBIC) of 6.25 mg/ml. AlKanderi et al. (150) further reported that stevia reduced biofilm formation and extracellular polysaccharide (EPS) production by *S. mutans* and *Streptococcus gordonii* (*S. gordonii*) and downregulated the expression of gtfB and gbpB, which are genes related to EPS synthesis. These findings suggest that stevia may reduce cariogenic risk by interfering with bacterial adhesion and biofilm matrix formation.

Human randomized controlled trials also support stevia's low cariogenic potential. Urrutia-Espinosa et al. (153) showed that, compared with sucrose, stevia had a smaller effect on oral pH reduction and bacterial activity, suggesting that its use as a sucrose substitute may help reduce acid production and caries-related risks. Pallepati et al. found that salivary pH returned to baseline within 1 h after consumption of stevia-sweetened tea, whereas pH remained below baseline in the sucrose and raw sugar groups (154). This suggests that stevia may have a lower cariogenic potential when used as a sweetener in tea beverages.

It should be noted that some commercial sweeteners containing stevia or aspartame may show cariogenicity similar to that of sucrose in microecological biofilm models, possibly due to the presence of lactose, bulking agents, or other fermentable excipients. In contrast, purified steviol glycoside sweeteners generally do not show obvious cariogenicity (155).

Therefore, the actual low-cariogenic effects require further validation in relation to the real oral environment, salivary systems, food matrices, excipient composition, and long-term intervention studies.

### 8.6 Potential benefits for gut microbiota and barrier function

The gut microbiota contributes to nutrient utilization, energy metabolism, intestinal barrier maintenance, and immune homeostasis through microbial metabolites and host–microbe signaling pathways, including short-chain fatty acids (SCFAs), bile acids (BAs), gut hormone signaling, and LPS-related inflammatory responses (156). In early life, the composition and function of the gut microbiota undergo dynamic changes influenced by age, delivery mode, geographical location, and gastrointestinal symptoms (157). Therefore, the effects of SGs as NNSs on the gut microbiota and intestinal barrier function are important considerations when evaluating their value in low-sugar foods.

Animal studies have shown that the bioactive components of stevia can improve gut microbiota dysbiosis induced by a high-fat, high-fructose diet, reduce the Firmicutes/Bacteroidetes ratio, and increase the abundance of *Lactobacillus* and *Akkermansia*, which are associated with improved intestinal mucosal barrier integrity and metabolic homeostasis (158). Individual glycosides, such as Reb A, can increase the abundance of *Bacteroides thetaiotaomicron* and alter Lactobacillus-related microbial structures, suggesting compound-specific effects among different glycosides (105). In addition, SGs can be hydrolyzed by the gut microbiota to steviol in the intestine. Although steviol itself is not sweet and is not the final sweetening component, it may participate in metabolic regulation and microbiota-related effects as an intestinal metabolite (17).

Stevia-derived constituents may support intestinal barrier function through antioxidant and anti-inflammatory mechanisms. Stevia residue extract improved intestinal morphology and oxidative stress-related biomarkers in hyperuricemic mice (159). In a diquat-induced IPEC-J2 cell model, stevioside reduced ROS and MDA levels, enhanced antioxidant enzyme activities, upregulated tight junction proteins, and suppressed IL-6, IL-8, TNF-α, and NF-κB/MAPK signaling, thereby improving epithelial barrier integrity (160).

Short-term human studies have shown that consuming stevia beverages among healthy adults does not lead to adverse changes in glucose tolerance, body mass index (BMI), or fasting metabolic markers. Some studies suggest that these neutral or potentially favorable effects may be related to improvements in the gut microbiome composition (161, 162).

Overall, SGs and related bioactive components may exert potential gut-related benefits by modulating gut microbiota composition, reducing oxidative stress and low-grade inflammation, and maintaining intestinal epithelial tight junctions and barrier integrity. However, the current evidence is not entirely consistent, and some NNSs may affect the gut microbiota and metabolic processes (102, 161). Therefore, the long-term effects of their intake still require careful attention.

Future studies should distinguish the effects of stevioside, Reb A, Reb M, and complex stevia extracts, and integrate real food matrices, long-term human interventions, and multi-omics analyses to further clarify their effects on intestinal barrier function, microbiome activity, and metabolic health.

### 8.7 Human evidence on stevia-derived sweeteners

**Figure 3:**
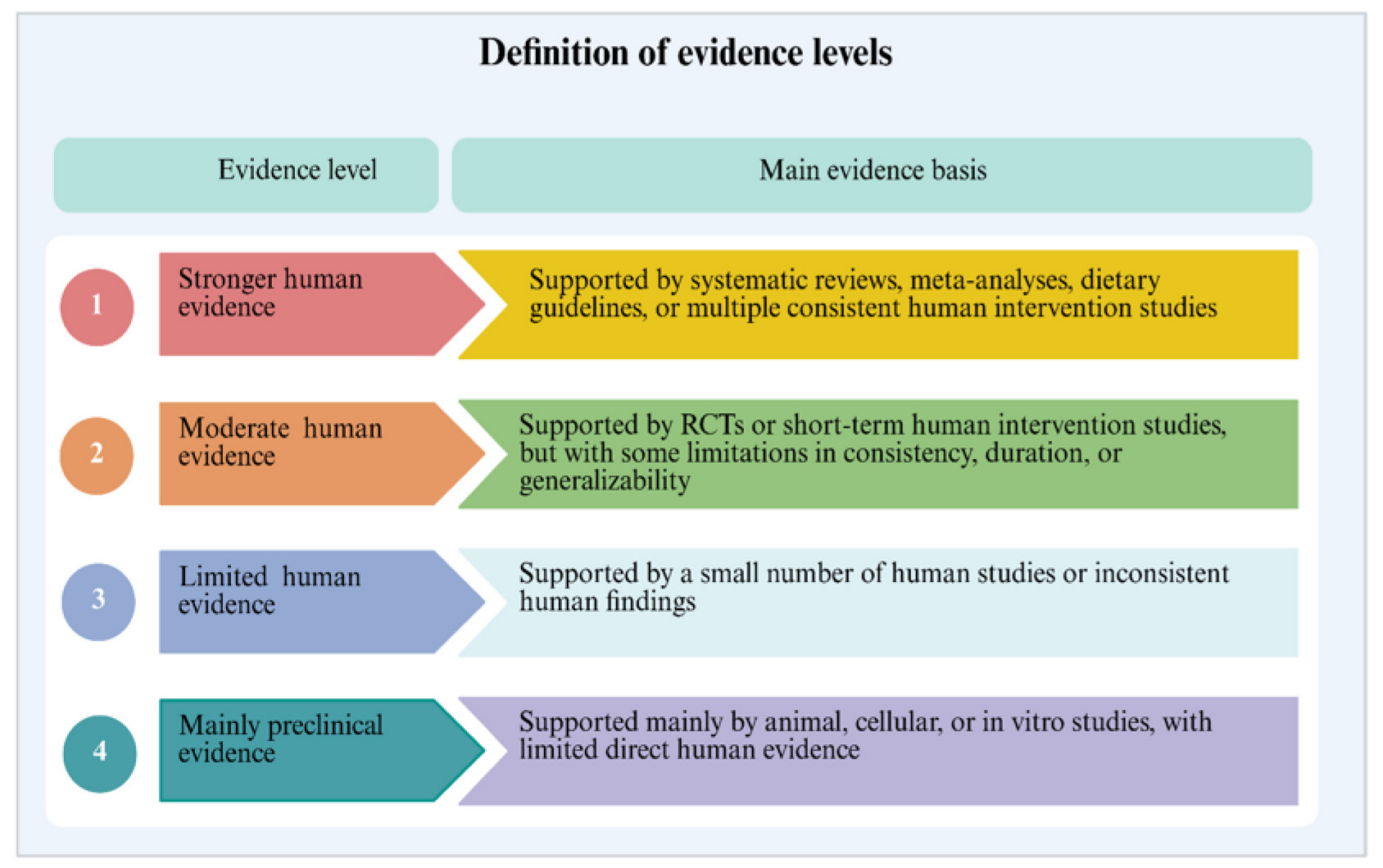
Evidence-level definitions for nutritional and metabolic effects of stevia.

Current human studies on Stevia-derived sweeteners have mainly focused on blood pressure, glucose metabolism, appetite and energy intake, gut microbiota, and safety and tolerability, whereas direct human evidence regarding antioxidant activity, anti-inflammatory effects, lipid metabolism, and intestinal barrier protection remains limited. To facilitate a more cautious stratified interpretation of the different types of nutritional evidence, Figure 3 defines the evidence levels adopted in this review, and Table 5 provides a narrative summary of the relevant nutritional evidence according to these levels. Table 6 summarizes information on human clinical trials of stevioside products.

**Table 6:** Summary of human evidence on stevia-derived sweeteners and related products.

Study design	Population	Stevia-derived intervention	Dose	Comparator	Duration	Main findings	Evidence implication	Limitations	Ref.
Randomized, triple-blind, parallel-controlled trial	Overweight adults	Stevia-sweetened citrus-maqui beverage	330 ml/day	Sucralose- or sucrose-sweetened beverage	60 days	The stevia group showed increased interleukin-10 (IL-10); oxygen radical absorbance capacity (ORAC) improved in participants with a low baseline antioxidant status, and no increase in homocysteine was observed.	Supports the possibility that a stevia beverage in a real food matrix may exert mild effects on oxidative stress- and inflammation-related markers.	Composite fruit-juice matrix; effects cannot be fully attributed to stevia alone.	(226)
Multi-arm randomized controlled trial	Healthy adults	Stevia-containing non-nutritive sweetener (NNS)	Commercial NNS supplement	Glucose vehicle or no supplement	2 weeks	Altered oral and gut microbiome profiles and plasma metabolome; the stevia group did not show group-level impairment of glucose tolerance.	Suggests a relatively mild effect of stevia on glucose tolerance, although microbiome responses may be individualized.	Short duration; commercial excipients may influence the results; long-term relevance remains unclear.	(161)
Randomized, double-blind, parallel-controlled trial	Healthy adults	SGs beverage	25% of the acceptable daily intake (ADI)	30 g sucrose beverage	4 weeks	No significant changes were observed in the gut microbiome composition, short-chain fatty acids (SCFAs), fasting glucose, insulin, lipids, or blood pressure; the sucrose group showed a slight increase in BMI.	Supports that moderate short-term intake of SGs does not markedly disturb gut microbiota or metabolic markers in healthy adults.	Healthy participants, short duration, and industry funding should be considered.	(162)
Randomized controlled trial	Dental students	Stevia	Oral exposure	D-tagatose or sucrose	Short-term	Compared with sucrose, stevia produced a smaller decrease in oral pH and a smaller increase in bacterial activity.	Provides short-term human evidence supporting stevia as a low-cariogenic sweetener alternative.	Small sample size, short observation time, population limited to dental students.	(153)
Randomized, controlled, open-label, two-parallel-arm trial	Healthy normal-weight adults	Commercial stevia drops containing stevia leaf extract in water	5 drops twice daily with habitual drinks	Control group maintaining usual diet without additional intervention	12 weeks	Daily stevia intake did not significantly affect oral glucose tolerance test (OGTT) glucose or insulin responses in the oral glucose tolerance test; the stevia group maintained body weight, whereas the control group gained weight. Self-reported energy intake decreased in the stevia group.	Supports a generally neutral effect on glycemic homeostasis and suggests potential benefits for moderating energy intake and weight maintenance under real-life use conditions.	An open-label design, no placebo control, small sample size, healthy normal-weight adults, self-reported energy intake, body weight, and energy intake were secondary outcomes.	(125)
Randomized, double-blind, placebo-controlled, parallel-group, repeated-exposure study	76 participants, including patients with type 1 diabetes, patients with type 2 diabetes, and non-diabetic participants with normal or low-normal blood pressure	SGs, mainly STV, purity **≥** 92%	250 mg three times daily	Placebo	3 months	No significant changes from baseline in systolic blood pressure (SBP), diastolic blood pressure (DBP), glucose, or hemoglobin A1c (HbA1c) were observed, and no hypoglycemia, hypotension, or obvious adverse effects were observed.	Supports good tolerability and metabolic neutrality of SGs at sweetener use doses; does not support clear pharmacological glucose- or blood pressure-lowering effects in these populations.	Small sample size; preliminary study; high variability in diabetic subgroups; mainly informs safety and absence of pharmacological effects rather than long-term real-food intake effects.	(193)
Multicenter, randomized, double-blind, placebo-controlled clinical trial	Patients with type 2 diabetes mellitus	Reb A, 97% purity, capsule form	1,000 mg/day; four 250 mg capsules daily, taken with morning and evening meals	Placebo capsules	16 weeks	No significant differences between the Reb A and placebo groups in changes in HbA1c, fasting glucose, insulin, C-peptide, blood pressure, body weight, or fasting lipids; no increase in hypoglycemic events; and good overall tolerability.	Supports metabolic neutrality and safety of long-term Reb A intake in patients with type 2 diabetes; does not support a clear glucose- or blood pressure-lowering pharmacological effect.	Participants had relatively well-controlled type 2 diabetes; capsule intervention rather than real food matrix, Cargill-related involvement, and provision of Reb A should be considered.	(213)
Multicenter, randomized, double-blind, placebo-controlled trial	Chinese patients with mild essential hypertension	STV capsules	500 mg three times daily; total 1500 mg/day	Placebo capsules	2 years	In the STV group, SBP decreased from 150 to 140 mmHg and DBP decreased from 95 to 89 mmHg, with significant reductions compared to baseline and placebo; quality-of-life scores improved; no significant laboratory abnormalities or serious adverse events were observed; left ventricular mass index (LVMI) was not significantly changed, but the incidence of left ventricular hypertrophy (LVH) was lower than in the placebo group.	Supports the long-term antihypertensive potential and tolerability of high-dose stevioside in mild hypertension.	Disease population; dose far exceeding typical food sweetener intake; capsule administration rather than real food matrix; conclusions should not be directly extrapolated to healthy populations or routine low-sugar food consumption.	(222)
Prospective, randomized, double-blind, placebo-controlled, single-center clinical trial	Untreated patients with mild essential hypertension	Crude STV derived from stevia leaves, mainly a mixture of STV and Reb A	Stepwise escalating dose: 3.75 mg/kg/day for 7 weeks, 7.5 mg/kg/day for 11 weeks, and 15.0 mg/kg/day for 6 weeks; twice daily	Placebo capsules	4-week placebo run-in plus 24-week intervention	SBP and DBP decreased in both the stevioside and placebo groups, but between-group differences were not significant; the highest dose of 15 mg/kg/day did not show a clear antihypertensive effect; and no major adverse events or obvious abnormalities in laboratory, electrocardiogram (ECG), or sex hormone-related indices were observed.	Suggests that crude stevioside did not show a placebo-independent antihypertensive effect in mild hypertension, although tolerability was good.	Very small sample size; single-center design; dose exceeding the ADI; relatively low baseline blood pressure; twice-daily dosing differed from previous studies; crude stevioside is compositionally complex and not equivalent to purified stevioside or Reb A.	(227)
Randomized parallel-controlled trial	Adults with overweight or obesity	Reb A-sweetened beverage	1.25–1.75 L/day Reb A-sweetened beverage; < 5 kcal/day	Sucrose-, aspartame-, saccharin-, or sucralose-sweetened beverages	12 weeks	The sucrose and saccharin groups showed significant increases in body weight; the Reb A group showed no significant weight gain; and no significant effects on glucose tolerance were observed across sweetener groups.	Supports that replacing sucrose with Reb A may help avoid sucrose-associated weight gain; different low-calorie sweeteners should not be treated as homogeneous interventions.	Overweight/obese population; large beverage volume; beverage-based intervention rather than typical food matrices; sweet taste; metabolic and behavioral effects may differ across low-calorie sweeteners.	(215)
Randomized pre-post preliminary human study	Young healthy adults with normal BMI	Commercial SGs	Four sachets/day; 1 g per sachet, total 4 g/day	Sucrose or sucralose	6 weeks	No significant pre-post changes in neuropsychological tests or electroencephalography/ quantitative electroencephalography (EEG/qEEG) indices were observed in the steviol glycoside group. The sucralose group showed declines in memory and executive function, along with changes in theta waves.	Preliminary evidence supports the absence of obvious short-term adverse central nervous system effects following steviol glycoside intake.	Small sample size; preliminary study; young healthy adults; commercial sweetener formulation may contain excipients; findings cannot be extrapolated to long-term neurological safety.	(228)
Randomized, double-blind, crossover-controlled trial	Healthy adults	Stevia-sweetened beverage	330 ml beverage containing 240 ppm stevia	Water, glucose beverage, sucrose beverage, or maltodextrin beverage	Acute premeal intervention	The stevia beverage did not increase blood glucose, total energy intake was lower than in the water condition, and attentional bias to food cues was not altered.	Supports that premeal stevia beverage consumption may acutely reduce appetite and total energy intake without increasing glycemia.	Acute study; small sample size; healthy normal-weight participants; not representative of long-term body weight management.	(217)
Randomized crossover acute trial	Women with insulin resistance and type 2 diabetes	Stevia preload	15.3 mg stevia dissolved in 60 ml water; consumed 10 min before glucose load	Water or D-tagatose	Acute; 3-h OGTT observation	The stevia condition showed a higher C-peptide incremental area under the curve (iAUC) than D-tagatose and water; glucose levels at 30 and 60 min were higher than in other conditions; subjective satiety increased at some time points, but objective food intake did not differ significantly.	Suggests that stevia may induce acute beta-cell/glucose responses in insulin-resistant women and should not be described as completely metabolically inert.	Acute study; only women with insulin resistance; dose and administration form differ from daily food intake; long-term metabolic effects cannot be inferred.	(220)
Multicenter prospective cohort study	Children and adolescents aged 7–17 years in Taiwan	Multiple sweeteners, including SGs	Food frequency questionnaire (FFQ) assessment of NNS intake combined with urinary biomarkers	Different sweetener intake levels	Observational follow-up	Aspartame and added sugars were associated with an increased risk of pediatric hypertension; SGs showed a marginal protective trend; replacing added sugar with NNSs was associated with a lower hypertension risk.	Suggests that different sweeteners may have different associations with pediatric blood pressure risk; SGs did not show a signal of increased hypertension risk.	Observational study; cannot establish causality; dietary intake measurement error; findings in children cannot be directly extrapolated to adults.	(223)
Three-arm randomized crossover trial	Healthy adults	Stevia extract preload	1 g stevia preload	Water or 60 g sugar preload	Acute premeal intervention	The stevia group reported lower hunger and a lower desire to eat than the water group; *ad libitum* meal intake and total daily energy intake were not significantly different; postprandial glucose did not increase further.	Suggests that acute stevia preload may reduce subjective appetite without increasing postprandial glycemia, although effects on total energy intake were limited.	Acute study; healthy adults; small sample size; relatively high stevia dose and may not represent long-term real food intake.	(218)
Randomized, placebo-controlled, open-label, two-way crossover pharmacokinetic trial	Patients with type 2 diabetes mellitus	Reb A, purity ≥97%	Single oral dose of 3 g	Placebo	Acute; OGTT conducted 19 h after dosing	Reb A was detectable and metabolized to steviol and steviol glucuronide; however, during OGTT, glucose area under the curve (AUC), insulin, and C-peptide did not differ significantly from placebo.	Supports that a single high dose of Reb A did not show a direct glucose-lowering effect in patients with type 2 diabetes.	Acute single high-dose study; capsule form; open-label design; not representative of routine low-sugar food intake.	(214)
Randomized crossover trial	Healthy participants	Biscuits containing stevia leaf powder	Biscuits containing 3% stevia leaf powder; each serving provided 50 g available carbohydrate	Plain wheat biscuits or moringa biscuits	Acute breakfast trial	Stevia biscuits did not significantly reduce postprandial glucose, but reduced hunger; palatability was acceptable, and no obvious gastrointestinal discomfort was observed.	Supports that stevia leaf powder in a real food matrix may improve subjective hunger, although the glucose-lowering effects were not clear.	Intervention used leaf powder rather than purified SGs; small sample size; acute study; biscuit matrix may strongly influence responses.	(221)
Double-blind, multicenter, randomized crossover acute trial	Adults with overweight or obesity	Blended sweetener beverages: Reb M + mogroside V; Reb A + thaumatin	330 ml zero-energy sweetened beverage	8% sucrose beverage	Acute; 4-h postprandial observation	All sweetener blend groups reduced 2-h insulin iAUC; the Reb A blend reduced glucose iAUC; appetite scores changed slightly but did not translate into differences in 24-h energy intake; and gastrointestinal symptoms were mostly mild.	Supports that stevia-containing blended beverages can reduce postprandial glucose and insulin responses compared with sucrose, while their effects on appetite and energy intake appear limited.	Acute study; blended sweeteners preclude attribution to a single stevia constituent; participants were adults with overweight or obesity.	(210)
Randomized crossover trial	Healthy adults	Sucrose/stevia blended cola beverage	355 ml beverage	Carbonated water, aspartame/acesulfame beverage, or sucrose beverage	Acute; 120-minute observation	The sucrose beverage induced the highest glucose and insulin responses; the sucrose/stevia beverage produced lower glucose levels at 60 min and increased pancreatic polypeptide (PP) levels.	Suggests that stevia-sucrose blended beverages may modify postprandial glucose and enteroendocrine hormone responses.	Composite sweetened beverage; effects cannot be attributed to stevia alone; small sample size; acute study.	(211)
Randomized crossover trial	Healthy men	Stevia-sweetened beverage	500 ml single preload	Sucrose-, aspartame-, or monk fruit-sweetened beverage	24-h continuous glucose monitoring	No significant differences were observed among stevia, monk fruit, aspartame, and sucrose beverages in 24-h mean glucose, iAUC, total area under the curve (AUC), or glycemic variability.	Suggests that single stevia beverage substitution for sucrose has limited effects on the 24-h glycemic profile in healthy men.	Very small sample size; healthy men only; single beverage substitution; not representative of long-term metabolic effects.	(54)
Randomized crossover trial	Healthy normal-weight men (n = 30)	Stevia-sweetened beverage, mainly Reb A	500 ml beverage containing 0.33 g stevia	Sucrose-, aspartame-, or monk fruit-sweetened beverage	Acute; 3-h postprandial observation and daily energy intake assessment	The NNS groups had higher *ad libitum* lunch intake than the sucrose group, but total daily energy intake did not differ significantly; the sucrose group had higher early glucose and insulin peaks, although the 3-h total AUC did not differ significantly.	Suggests that single stevia beverage substitution for sucrose can reduce the beverage sugar load, but subsequent energy compensation may occur; overall postprandial glucose and insulin AUC differences were limited.	Healthy men only; acute study; not representative of long-term weight or metabolic effects; predominant liquid beverage matrix.	(229)
Randomized, double-blind, crossover trial with acute and 2-week repeated-intake phases	Adults with overweight or obesity	Stevia rebaudioside M-sweetened cookies (StRebM)	Daily intake of StRebM-reformulated cream-filled cookies	Sucrose cookies or neotame cookies	Acute and 2 weeks for each formulation	StRebM and sucrose did not differ significantly in appetite ratings or ghrelin, glucagon-like peptide 1 (GLP-1), and PP responses; StRebM reduced postprandial insulin and glucose responses compared with sucrose.	Replacing sucrose with StRebM in solid foods can reduce postprandial glucose and insulin responses without evidence of increased appetite.	Short intervention duration; adults with overweight or obesity; evaluated a specific cookie matrix and StRebM only; not all SGs or long-term body weight effects.	(212)
Randomized, controlled, single-blind preliminary human trial	Healthy adults	SUITENA(TM), a blended sugar substitute containing erythritol, xylitol, and stevia	50 g SUITENA™ dissolved in 250 ml water	50 g dextrose monohydrate dissolved in 250 ml water	Acute; blood glucose measured over 90 min	SUITENA™ produced a significantly lower glycemic response than dextrose; its mean AUC was 15.31 mmol/L/90 min compared with 177.25 mmol/L/90 min for dextrose, corresponding to 8% of the glycemic response of the standard	Suggests that this polyol–stevia blended sugar replacer elicits a low acute glycemic response in healthy adults	Very small sample size; acute study; healthy adults only; product was a blend of erythritol, xylitol, and stevia, so the effect cannot be attributed to stevia alone; funded by the product developer	(115)

ADI, acceptable daily intake; AUC, area under the curve; BMI, body mass index; DBP, diastolic blood pressure; GLP-1, glucagon-like peptide 1; HbA1c, hemoglobin A1c; iAUC, incremental area under the curve; NNS/NNSs, non-nutritive sweetener(s); OGTT, oral glucose tolerance test; PP, pancreatic polypeptide; Reb A, rebaudioside A; Reb M, rebaudioside M; SBP, systolic blood pressure; SCFAs, short-chain fatty acids; SGs, steviol glycosides; StRebM, stevia rebaudioside M.

This table summarizes human evidence on stevia-derived sweeteners and related products, focusing on the study design, population, intervention, dose, comparator, duration, main findings, evidence implications, and limitations. Because studies differ in steviol glycoside type, dose, food matrix, participant characteristics, and intervention duration, the findings should be interpreted in relation to specific study conditions.

## 9 Comparative positioning of stevia in low-sugar sweetener systems

Commonly used NNSs can be broadly classified as synthetic or naturally derived sweeteners. The FDA has approved six synthetic high-intensity sweeteners as food additives, including aspartame, acesulfame potassium, saccharin, sucralose, neotame, and advantame. In addition, the FDA has not questioned the generally recognized as safe (GRAS) determinations for certain high-purity SGs and monk fruit extracts under their intended conditions of use (163, 164).

### 9.1 Comparison with artificial non-nutritive sweeteners

Stevia-derived sweeteners are generally perceived as natural and are characterized by high sweetness intensity, low energy contribution, and a relatively well-established safety evaluation basis (19). However, concerns remain regarding the long-term, high-dose intake of some synthetic sweeteners, as some studies have suggested possible associations with metabolic alterations, including impaired vascular endothelial function, increased fat storage, and reduced insulin sensitivity (12). Some observational studies have also reported associations between artificial sweetener intake and increased risks of cardiovascular events and mortality (165). Potential mechanisms may involve changes in the gut microbiota, endothelial dysfunction, insulin resistance, inflammatory responses, and alterations in cardiovascular risk factors (166, 167). However, these observational associations should be interpreted with caution and not considered causal evidence. In addition, taking aspartame as an example, the International Agency for Research on Cancer (IARC) classified it as Group 2B, namely “possibly carcinogenic to humans.” In contrast, JECFA reaffirmed its acceptable daily intake (ADI) of 0–40 mg/kg body weight per day (168).

Given the distinction between hazard identification and exposure-based risk assessment, the long-term health effects of NNSs should be interpreted by considering sweetener type, dose, exposure level, intake relative to the ADI, population characteristics, dietary context, and the type of evidence. Table 7 summarizes the differences between stevia-derived sweeteners and major artificial NNSs, providing a reference for sweetener selection in reduced-sugar food development.

**Table 7:** Comparison of stevia and commonly used synthetic non-nutritive sweeteners.

Sweetener	Relative sweetness vs. sucrose	Regulatory status	ADI (mg/kg bw/day)	Metabolism and absorption	Potential pharmacokinetic effects	Advantages in food applications	Limitations in food applications	Ref.
Saccharin	200–700×	U.S. Food and Drug Administration (FDA)-approved food additive; initially listed as generally recognized as safe (GRAS) in 1959 and regulated as a food additive in 1977.	5	Approximately 85% of saccharin is absorbed after oral intake and is mainly excreted unchanged; absorbed saccharin is highly bound to plasma proteins.	May affect cytochrome P450 (CYP) enzyme activity, including CYP1A1, CYP2E1, and CYP2D6 activity, reduce monoamine oxidase A (MAO-A) activity, and influence plasma albumin binding.	Low cost, high sweetness intensity, and good heat stability.	May have a metallic or bitter aftertaste; historical controversy regarding bladder cancer; potential pharmacokinetic interactions should be considered during concomitant drug use.	(185)
Aspartame	~200×	FDA-approved food additive	50	Hydrolyzed by intestinal enzymes into aspartic acid, phenylalanine, and methanol before absorption; a small fraction has been reported to be absorbed intact, although this remains uncertain.	May inhibit organic anion transporter 4 (OAT4) and peptide transporter 1 (PEPT1), affect CYP expression/activity, and bind to plasma proteins.	Sucrose-like sweetness with minimal aftertaste; widely used in beverages and foods.	Heat-labile and unsuitable for baking; phenylalanine-containing degradation products pose risks for individuals with phenylketonuria (PKU); historical health concerns remain debated.	(185)
Acesulfame potassium (Ace-K)	~200×	FDA-approved food additive	15	Almost completely absorbed after oral intake and is mainly excreted unchanged, with no evidence of metabolism.	May act as a P-glycoprotein (P-gp) substrate and competitive inhibitor and may bind to human serum albumin (HSA) and bovine serum albumin (BSA).	High sweetness intensity, good heat stability, and suitability for blending with other sweeteners to improve taste.	A mild bitter aftertaste may occur at high concentrations, and potential drug interactions should be considered when used as a pharmaceutical excipient.	(185)
Sucralose	~600×	FDA-approved food additive	5	Approximately 15% is absorbed intact, and a small fraction is recovered as glucuronide conjugates, indicating low rates of hepatic metabolism.	It may act as a P-gp substrate and competitive inhibitor; animal studies suggest possible upregulation of CYP3A4 and CYP2D1, whereas human liver microsome data did not show increased CYP3A4 activity; it may bind strongly to plasma albumin, affecting drug distribution and metabolism.	High sweetness intensity, clean taste, good heat stability, and broad applicability.	The potential effects on the gut microbiota remain debated; pharmacokinetic effects require further validation in humans.	(185)
SGs	200–400×	FDA: no questions on GRAS notices for certain high-purity SGs	4	Hydrolyzed by the gut microbiota to steviol, which is absorbed, metabolized in the liver to steviol glucuronide (SVG/SVAG), and excreted mainly in the urine.	Steviol may interact with organic anion transporting polypeptide (OATP) and organic anion transporter (OAT) transporters; OAT3 likely transports steviol glucuronide and may inhibit CYP2C8; steviol may induce CYP1A2, CYP1A1, CYP1B1, CYP3A4, and CYP2B6 expression/activity and may affect CYP3A4/CYP2C9 substrate metabolism; and SGs and metabolites may bind to BSA.	Natural origin and high sweetness intensity; commonly perceived as a healthier sugar substitute.	At high concentrations, licorice-like or bitter aftertastes may occur, and metabolites may interact with drug transporters and enzymes, requiring cautious evaluation of potential effects.	(185)

Ace-K, acesulfame potassium; ADI, acceptable daily intake; BSA, bovine serum albumin; bw, body weight; CYP, cytochrome P450; FDA, U.S. Food and Drug Administration; GRAS, generally recognized as safe; HSA, human serum albumin; MAO-A, monoamine oxidase A; OAT, organic anion transporter; OAT3, organic anion transporter 3; OAT4, organic anion transporter 4; OATP, organic anion transporting polypeptide; PEPT1, peptide transporter 1; P-gp, P-glycoprotein; PKU, phenylketonuria; Ref., reference; SGs, steviol glycosides; SVG/SVAG, steviol glucuronide.

Potential pharmacokinetic effects are derived mainly from *in vitro*, animal, or limited mechanistic studies and should not be interpreted as confirmed food-drug interactions in humans under normal dietary exposure.

### 9.2 Comparison of stevia with other naturally derived sweeteners

Naturally derived sweeteners, such as monk fruit extract, thaumatin, miraculin, brazzein, and monellin, have also been used in the development of sugar-reduced foods (19, 169–174). Notably, although these sweeteners are derived from natural sources, some compounds, such as miraculin and mogroside V, may affect intestinal epithelial function via T1R3-mediated pathways, similar to those reported for synthetic sweeteners such as saccharin (175). These effects may include the induction of oxidative stress, disruption of barrier integrity, and regulation of genes related to cellular junctions (175).

Table 8 systematically compares different naturally derived sweeteners in terms of sweetness intensity, metabolism, absorption characteristics, major advantages and limitations, and typical food applications.

**Table 8:** Comparison of stevia and other naturally derived sweeteners in low-sugar foods.

Sweetener category	Relative sweetness vs. sucrose	Regulatory status	Metabolism and absorption characteristics	Main advantages	Main limitations	Typical food applications	Ref.
SGs	200–350×	Generally recognized as safe (GRAS) for certain high-purity SGs	Hydrolyzed by gut microbiota to steviol, which is subsequently metabolized in the liver to steviol glucuronide (SVG) and excreted.	High sweetness intensity and low energy contribution; high-purity SGs have a relatively mature safety and regulatory evaluation basis.	May cause bitterness, licorice-like aftertaste, and lingering sweetness; when used alone, they cannot fully reproduce the sensory properties of sucrose and usually require blending or taste-masking strategies.	Beverages, dairy products, bakery products, confectionery, jams, tabletop sweeteners, and blended sugar-reduction systems.	(13, 19, 24, 25)
Mogrosides	100–250×	GRAS for certain monk fruit extracts	Glycosylated mogrosides are poorly absorbed and hydrolyzed by the gut microbiota to mogrol; mogrol and its metabolites can be absorbed.	High sweetness intensity, low energy contribution, mild bitter aftertaste, and plant-derived origin; can be blended with SGs to optimize sweetness profiles.	Limited evidence exists regarding individual mogroside composition, sensory mechanisms, long-term human metabolism, and standardized food applications. Some *in vitro* studies suggest that high concentrations of mogroside V may affect intestinal epithelial barrier function.	Premium tea beverages, herbal beverages, functional foods, natural-label beverages, and blended sweetener systems.	(19, 169, 175, 185)
Thaumatin	2000–3000×	U.S. Food and Drug Administration (FDA) GRAS; evaluated by the European Food Safety Authority (EFSA) and authorized in the European Union as food additive E 957.	Rapidly digested like dietary proteins, generally considered non-toxic and non-allergenic.	Natural sweet-tasting proteins with extremely high sweetness intensity can enhance sweetness and mask undesirable flavors.	Sensitive to processing conditions, especially high temperature and pH > 7; slow onset of sweetness; may produce a licorice-like aftertaste at high concentrations; relatively high cost.	Flavor enhancer, sweetness modifier, acidic foods, premium desserts, beverages, and selected functional foods.	(170, 230, 231)
Brazzein	500–2000×	Under research and development; regulatory approval remains limited	Protein-based sweeteners that are digested into amino acids.	High sweetness intensity and good flavor potential; high thermal stability, good solubility, and favorable mouthfeel.	Low degree of industrialization; limited evidence from human studies, long-term safety evaluation, regulatory pathways, and applications in real food matrices.	Potential future use in premium sweetener systems or specialized formulations.	(171, 172)
Monellin	~3000×	Under research and development; not yet widely industrialized	Protein-based sweeteners that are digested into amino acids.	High sweetness intensity and clean taste can markedly enhance sweetness at low concentrations.	Sensitive to temperature and pH; denatures and loses sweetness when heated above 50 °C, with poor processing stability.	Premium sweetener products and specialized functional foods.	(171, 172)
Miraculin	Taste-modifying protein	Under research and development	Not a direct sweetener; induces sweetness perception under acidic conditions.	It can induce sweetness perception in acidic environments and is suitable for designing special taste experiences.	Not a typical direct sweetener; application is limited by pH conditions; long-term human evidence and food application data remain limited; and *in vitro* studies suggest that intestinal epithelial effects should be considered.	Taste-modifying products, special sensory-experience foods, and acidic food systems.	(173–175, 232, 233)

SGs, steviol glycosides; EFSA, European Food Safety Authority; FDA, U.S. Food and Drug Administration; GRAS, generally recognized as safe; Ref., reference; SVG, steviol glucuronide.

From the perspective of sugar-reduced food development and practical application, stevia shows comparative advantages in terms of application maturity and safety evaluation, and may serve as an important option in sugar-reduction strategies (161, 176).

## 10 Regulatory status of steviol glycosides

Sweeteners derived from *Stevia rebaudiana* Bertoni have been widely used in the development of reduced-sugar foods. However, in food regulatory contexts, “stevia” is not a single ingredient category, but may refer to purified SGs, stevia leaves, crude stevia extracts, enzyme-modified SGs, fermentation-derived SGs, glucosylated SGs, or other stevia-derived ingredients (39, 177). Because these materials differ in composition, purity, production route, and extent of toxicological evaluation, distinguishing their regulatory status is important to avoid inappropriate extrapolation of safety conclusions from high-purity SGs to less purified stevia-derived materials (39, 177–180).

At the international level, JECFA and EFSA provide major references for the safety assessment and specification of SGs. JECFA has established an ADI of 0–4 mg/kg body weight/day for SGs, expressed as steviol equivalents, and provides specifications for SGs obtained from different sources and production routes, including leaf extraction, fermentation, enzymatic modification, and enzymatically modified glucosylation (39, 177). These specifications generally require at least 95% total SGs on a dried basis, with route-specific requirements for certain modified products (177). EFSA has also emphasized that more than 60 SGs identified in stevia leaves and sharing a common metabolic fate may be evaluated within a common safety framework (26), while non-glycosidic components and production-related impurities should still be adequately characterized and controlled (39, 181, 182).

Against this international safety assessment basis, national and regional frameworks still differ in regulatory pathway, ingredient definition, production process, authorized uses, maximum use levels, labeling requirements, and the regulatory status of stevia leaves or crude extracts (26, 39, 177, 183). In particular, the use of enzyme-modified and fermentation-derived SGs has shifted regulatory attention beyond conventional high-purity SGs toward the control of production-route-related residual proteins, residual DNA, by-products, and non-glycosidic components (26, 39, 177, 181, 184).

Table 9 summarizes the regulatory status of SGs and selected stevia-derived ingredients in major countries and regions, focusing on permitted ingredient types, purity or specification requirements, ADI, the regulatory status of stevia leaves or crude extracts, covered production routes, and key use restrictions. This comparison highlights that SG ingredient selection, use-level design, labeling claims, and cross-border marketing strategies in reduced-sugar food development should align with the specific ingredient type, production process, and regulatory requirements of the target market.

**Table 9:** Regulatory status of steviol glycosides and selected stevia-derived ingredients across international and regional frameworks.

Region or authority	Regulatory category	Applicable steviol glycoside type	Purity or specification requirement	ADI	Status of stevia leaves or crude extracts	Production routes	Key restrictions or notes	Reference
JECFA	International safety evaluation and food-additive specification reference for steviol glycosides (SGs); not a market authorization system.	Annex 1: SGs from Stevia rebaudiana Bertoni; Annex 2: fermentation-derived SGs; Annex 3: enzyme-modified SGs; Annex 4: enzyme-modified glucosylated SGs.	Generally not less than 95% total SGs on a dried basis; enzyme-modified glucosylated SGs are specified on a dried, dextrin-free basis.	ADI: 0–4 mg/kg bw/day, expressed as steviol equivalents.	Whole leaves and crude extracts are not addressed as permitted ingredient types	Leaf extraction, fermentation, enzymatic modification, and enzymatically modified glucosylation.	Provides internationally referenced identity, assay, purity, residual solvent, heavy-metal, and microbiological specifications; national authorization and use conditions remain subject to local regulations.	(39, 177, 234, 235)
United States/FDA	Specific high-purity SG preparations may be used when they are the subject of GRAS notices that have received FDA “no questions” responses; the responses are substance- and use-specific.	GRAS notices include purified SGs such as Reb A, Reb D, and Reb M, as well as enzyme-modified, glucosylated, and fermentation-derived preparations.	GRAS notices often specify ≥95% total.	0–4 mg/kg bw/day, expressed as steviol equivalents.	Stevia leaf and crude stevia extracts are not approved food additives and are not considered GRAS for use as sweeteners in conventional foods.	Leaf-derived purification, enzymatic modification, glucosylation, and fermentation, depending on the specific GRAS notice.	A “no questions” response should not be generalized to all stevia-derived materials, ingredient types, or use conditions.	(178, 179)
Canada/Health Canada, CFIA	Purified stevia extract and SGs are regulated as food additive sweeteners for specified foods; stevia and stevia extracts may also be used in natural health products (NHPs) under Health Canada conditions.	purified stevia extract/SGs, including plant-derived and authorized fermentation-derived forms where applicable. NHP context: stevia and SGs/stevia extracts as medicinal or non-medicinal ingredients, subject to NHP requirements.	For food-additive SGs, the total SGs content should be at least 95% on a dried basis and conform to FCC or JECFA specifications, where applicable.	0–4 mg/kg bw/day, expressed as steviol equivalents.	In foods, stevia leaves and crude extracts are treated differently from approved food-additive SG sweeteners and are considered food ingredients rather than food additive sweeteners.	Plant extraction and purification; authorized fermentation-derived SGs where specified by Health Canada/CFIA.	Food use is limited to permitted food categories, maximum permitted levels, and conditions of use. NHP use must comply with Health Canada NHP limits, labeling, and evidence requirements. Health Canada has not reached the same definitive safety conclusion for stevia leaves in retail foods as for purified food-additive SGs.	(180, 236, 237)
EU/EFSA, European Commission	SGs are authorized as E 960a-d food additive sweeteners. EFSA conducts risk assessment, whereas the European Commission adopts legal authorisations and specifications.	E 960a: SGs from stevia; E 960b: fermentation-derived SGs; E 960c: enzymatically produced SGs; E 960d: glucosylated SGs. The list of steviosides has been expanded to include more than 60 SGs identified in the leaves of Stevia rebaudiana Bertoni.	a steviol glycoside preparation must contain not less than 95% of SGs or subcategory-specific purity requirements.	4 mg/kg bw/day expressed as steviol equivalents.	Whole leaves and crude extracts should not be treated as equivalent to authorized SG additives.	Stevia leaf extraction, enzymatic production, glucosylation, and fermentation-derived production, including authorized Reb M from *Yarrowia lipolytica*.	Use is limited to authorized food categories, with maximum permitted levels of 20–3300 mg steviol equivalents/kg and quantum satis use for tabletop sweeteners. Enzymatically modified and fermentation-derived products require additional evaluation and route-specific control of non-glycosidic components, process-related impurities, pesticide-residue changes, heavy metals, and microbiological criteria.	(26, 181, 184)
China/NHC, SAMR, CFSA	SGs are permitted as food additive sweeteners under GB 2760. GB 1886.355-2022 sets specifications for food additive SGs, and enzyme-converted SGs are covered by NHC announcements.	Leaf-extracted SGs are under GB 1886.355-2022, and enzyme-converted SGs are under NHC announcements. Listed SGs include stevioside, rebaudiosides A, B, C, D, E, F, M, N and O, dulcoside A, rubusoside, and steviolbioside.	Generally not less than 95% total SGs on the dried basis	0–4 mg/kg bw/day, expressed as steviol equivalents.	Whole stevia leaves or crude extracts should not be treated as equivalent to approved food additive SGs.	Leaf extraction and refining; approved enzyme-conversion routes for specific SG products.	GB 2760-2024 specifies permitted food categories and maximum use levels. GB 1886.355-2022 specifies sensory, physicochemical, and safety criteria. Enzyme-converted SGs are authorized for specified categories, including modified milk, flavored fermented milk, and gum-based confectionery. Stevia polyphenols are regulated separately as a new food raw material, with a recommended intake not exceeding 500 mg/day.	(238–241)
Japan/CAA, MHLW	Stevia-related ingredients are listed as existing food additives under Japan's food additive framework.	Stevia extract, powdered stevia, SGs, and alpha-glucosyltransferase-treated stevia/SGs.	Generally not less than 95% total SGs on the dried basis	No separate Japan-specific ADI is listed for these existing additive entries; the JECFA ADI may be cited for international safety context.	Powdered stevia and stevia extract are listed as existing additives	Leaf extraction, leaf powdering, and alpha-glucosyltransferase treatment. No explicit fermentation-derived SG category is identified in Japan's official additive listings.	Products are regulated under Japan's existing-additive system and official food additive specifications	(242–244)
Republic of Korea/MFDS	Steviol Glycoside (INS 960) and Enzymatically Modified Stevia are listed as food additive specification items under Korea's Food Additives Code.	Leaf-derived SGs; enzymatically modified stevia/glucosyl stevia; and bioconversion-based SGs addressed by the MFDS 2025 amendment.	Steviol Glycoside: not less than 95.0% total SGs on a dried basis. Enzymatically Modified Stevia: not less than 80.0% SGs and not more than 15.0% unreacted SGs on a dried basis.	No separate ADI is specified.	The specifications apply to purified products made from dried stevia leaves or modified stevia extract, not to whole leaves or crude extracts.	Hot-water extraction from dried Stevia rebaudiana Bertoni leaves, resin adsorption/concentration, methanol or ethanol recrystallisation/purification, drying, enzymatic modification, alpha-glucosyltransferase treatment, and bioconversion under the MFDS 2025 amendment.	Products must comply with Korea's Standards and Specifications for Food Additives, including identity, assay, pH, heavy metals, residual solvents, and analytical requirements.	(245–247)
India/FSSAI	Steviol glycoside/INS 960 is permitted as a non-caloric sweetener food additive in specified food categories.	A mixture of SGs containing a steviol backbone and linked to sugars naturally present in the leaves of Stevia rebaudiana Bertoni.	Not less than 95% total SGs on a dried basis.	No separate ADI is specified.	Whole leaves/crude extracts should not be treated as equivalent to authorized SG additives.	Hot-water extraction, adsorption resin concentration, alcohol elution, recrystallisation, ion-exchange purification, and spray-drying.	FSSAI specifies requirements for pH, purity, residual solvents, heavy metals, and microbiological criteria. Use is limited to specified food categories and is currently not permitted in cocoa/chocolate products or imitation chocolate products.	(248, 249)
Australia and New Zealand/FSANZ	SGs (960) are permitted as intense sweetener food additives under the Australia New Zealand Food Standards Code.	SGs (960), including directly extracted, enzymatically converted, and fermentation-derived SGs where authorized; A1207 covers Reb M from *Saccharomyces cerevisiae* as a specific application.	Not less than 95% total SGs on a dried basis.	0–4 mg/kg bw/day, expressed as steviol equivalents.	The rules concern SG sweetener additives, not whole stevia leaves or crude extracts.	Direct extraction from stevia leaves; enzymatic conversion using enzymes from genetically modified microorganisms; fermentation from sugar using genetically modified yeast.	Use is limited to permitted food categories and maximum permitted levels. Labeling may use “sweetener (960)” or “sweetener (steviol glycosides)”.	(183, 250)

ADI, acceptable daily intake; bw, body weight; CAA, Consumer Affairs Agency of Japan; CFIA, Canadian Food Inspection Agency; CFSA, China National Center for Food Safety Risk Assessment; EFSA, European Food Safety Authority; FDA, U.S. Food and Drug Administration; FCC, Food Chemicals Codex; FSANZ, Food Standards Australia New Zealand; FSSAI, Food Safety and Standards Authority of India; GRAS, generally recognized as safe; INS, International Numbering System for Food Additives; JECFA, Joint FAO/WHO Expert Committee on Food Additives; MFDS, Ministry of Food and Drug Safety of the Republic of Korea; MHLW, Ministry of Health, Labor and Welfare of Japan; NHC, National Health Commission of the People's Republic of China; Reb, rebaudioside; SAMR, State Administration for Market Regulation; SGs, steviol glycosides.

“Quantum satis” means that no numerical maximum level is fixed, but the additive should be used in accordance with good manufacturing practice at a level no higher than necessary to achieve the intended technological effect. The table distinguishes purified SG food additive sweeteners from stevia leaves, crude extracts, and other stevia-derived ingredients such as stevia polyphenols.

## 11 Application scenarios of stevia

According to statistics from the U.S. Department of Agriculture (USDA), stevia is currently used as an ingredient in more than 7,000 branded food products (185). Supplementary material 1 summarizes representative Stevia-containing products and their key information available on major Chinese e-commerce platforms, including Taobao (https://www.taobao.com/) and JD.com (https://www.jd.com/), as well as the international e-commerce platform Amazon (https://www.amazon.com/). The products cover categories such as foods, beverages, dietary supplements, functional foods, health products, oral-care products, and selected medicines, providing a reference for understanding the application scenarios, product formats, and market layout of stevia in low-sugar product development.

## 12 Safety assessment of steviol glycosides

High-purity SGs have been supported by relatively systematic toxicological studies and regulatory evaluations. Major regulatory and authoritative assessment bodies consider specified high-purity SG preparations safe when used under approved conditions of use (26). SGs are not readily hydrolyzed by digestive enzymes in the upper human gastrointestinal tract. Instead, they are mainly converted by the gut microbiota to steviol, which is subsequently absorbed, metabolized, and primarily excreted in the urine, indicating low systemic accumulation (87, 182, 186). This metabolic profile appears to be generally consistent in both adults and children (187).

Under conventional exposure conditions, current genotoxicity assessments, long-term rodent carcinogenicity studies, and subchronic oral toxicity studies have not indicated mutagenic, chromosomal-damaging, carcinogenic, or significant organ-toxic effects of SGs (188–191).

However, the intake of SGs or Stevia-related products still requires cautious evaluation under specific conditions. High-purity SGs generally remove most plant-derived allergenic proteins, and allergic reactions are rare in the general population. Nevertheless, individuals with an allergy to Asteraceae or an atopic predisposition may be at risk of skin sensitization (192). Excessive intake of unrefined stevia leaves or crude stevia teas may lead to decreased blood pressure, bradycardia, or hypoglycemia (87, 182, 186, 193), although these effects have not been consistently reproduced in conventional sweetener intake studies. In addition, isolated case reports have suggested that stevia non-nutritive sweetener intake may be associated with restless legs syndrome-like symptoms, which resolved after discontinuation and reappeared after re-exposure (194).

Under non-conventional exposure conditions, some *in vitro* and animal studies have suggested potential risks associated with SGs or crude stevia extracts. For example, extremely high *in vitro* concentrations of SGs may induce DNA damage, chromosomal abnormalities, and inhibitory effects on immune cells (195, 196). Extreme-dose animal studies have also reported reproductive suppression and disturbances in immune homeostasis (197). However, these findings are mainly derived from high-concentration *in vitro* exposures, extreme-dose animal models, or unrefined crude extract models. They therefore should not be directly interpreted as safety risks for high-purity SGs under normal dietary exposure. In contrast, some mechanistic studies have suggested that, at appropriate concentrations, SGs may participate in reproductive physiological regulation through the T1R2 sweet taste receptor and GNAT3 signaling pathway, thereby exerting certain protective or improving effects on testicular tissue homeostasis and sperm quality in male animals (198, 199).

Overall, current evidence supports the consumption safety of high-purity SGs under conventional intake conditions. Nevertheless, further safety evaluation remains warranted for children, immunocompromised individuals, metabolically susceptible populations, and long-term high-frequency consumers, particularly through real food-matrix studies, long-term follow-up, and multi-omics approaches.

## 13 Conclusions and future perspectives

SGs occupy an important position in reduced-sugar food development because of their natural origin, high sweetness intensity, low energy contribution, and the safety evaluation and regulatory acceptance of specified high-purity SG preparations by major international authorities. However, in real food systems, SGs still cannot fully reproduce the flavor profile of sucrose, its functional roles in bulk, texture, water retention, browning reactions, and fermentation, or its reward-related effects. In addition, SGs are still associated with sensory limitations, including bitterness, lingering sweetness, licorice-like aftertaste, and insufficient oral fullness. To overcome these drawbacks, approaches including steviol glycoside blending, structural modification, enzymatic transformation, microbial fermentation, and compounding with sugar alcohols, rare sugars, dietary fibers, proteins, as well as polysaccharide carriers have exhibited great potential to enhance the sweet taste profile, oral texture, thermal processing stability, and formula compatibility of SGs. Future studies should further validate these approaches in real food systems containing SGs, with particular attention to standardized sensory evaluation, consumer preference testing, formulation-ratio optimization, storage stability assessment, and long-term safety validation under realistic consumption conditions, thereby improving their industrial applicability in reduced-sugar food development.

Notably, the differences between SGs and sucrose are not limited to oral sweetness perception, but also involve sweet and bitter taste receptors, extraoral sweet-taste signaling, and central reward responses. These mechanistic insights provide a basis for more precise formulation design using SGs. However, whether SGs can influence food preference, appetite regulation, reward-driven eating behavior, and long-term dietary adaptation under real-world dietary conditions remains insufficiently understood. Future studies should integrate sensory science, neuroimaging, and dietary behavior assessment to clarify the effects of SG-containing reduced-sugar foods on central reward responses and to determine whether they may help attenuate reward-driven preference for high-sugar foods.

Meanwhile, evidence regarding the potential health effects of SGs and other stevia-derived ingredients remains largely derived from *in vitro* experiments, animal studies, or short-term human trials. Future research should be guided by existing evidence grading. It should prioritize long-term randomized controlled trials, validation in real food matrices, multi-omics approaches, and population-specific studies to clarify the nutritional effects and reproducibility of different SGs, food matrices, and intake levels. For children, infants and toddlers, high consumers, and other potentially high-exposure populations, standardized intake and exposure assessments are needed to define the safety margins between total exposure under different consumption scenarios and the ADI.

Overall, SGs have broad application prospects in reduced-sugar foods, functional food design, and reduced-sugar dietary strategies. Future research should place greater emphasis on sensory optimization, validation in real food matrices, accumulation of human evidence, exposure and safety assessment, and regulatory alignment, thereby providing more direct scientific support for ingredient selection, formulation design, risk assessment, and cross-market application of SGs.
