# Supplementary material for: Stevia-derived sweeteners in reduced-sugar foods: formulation strategies, sensory optimization, nutritional evidence, and safety perspectives
Source: Front Nutr. 2026 Aug 5;13:1894194. doi: 10.3389/fnut.2026.1894194 (PMC13485817; doi:10.3389/fnut.2026.1894194)
Supplement: Supplementary file 1 [file Table_1.docx]

**Supplementary table 1. Representative stevia products available for purchase on major e-commerce platforms**

| **Category** | **Product Name** | **ASIN / Approval Number** | **Stevia-derived ingredient** | **Specification / Dosage** | **Manufacturer** |
| --- | --- | --- | --- | --- | --- |
| Food | Garden of Life Dr. Formulated Whole Food | B079D35ZKG | Organic Stevia Leaf Extract | 7 oz bottle | Garden of Life |
|  | Zollipops Clean Teeth Pops Sugar Free Candy | B094NWK4SP | Stevia | 5.2 oz bag | OTC Liquids, LLC |
|  | LILY'S Almond Dark Chocolate Style No Sugar Added | B07HL574CG | Stevia extracts | 3 oz Bar | The Hershey Company |
|  | HighKey Sugar Free Sandwich Cookies | B0BLPCSTL7 | Stevia Extract | 3.63 oz bag | HighKey |
|  | HighKey Cookies Variety Pack | B07ZTRTTY7 | Stevia Extract | 2.25 oz Pouch | HIGHKEY |
|  | Rip Van Dark Chocolate Wafer Cookies | B09BC3WF97 | Stevia Leaf Extract | 0.78 oz Wafer | Rip Van |
|  | Duncan Hines Keto Friendly Brownie Mix | B08VY7MX8X | Steviol glycosides | 10 oz box | Conagra |
|  | Catalina Crunch Protein Cereal | B07WD631ZX | Stevia Extract | 9 oz Bag | Catalina Crunch |
|  | Protein One Strawberries and Cream Protein Bars | B07BB3Y6BK | Stevia Extract | 0.96 oz bars | GENERAL MILLS |
| Drink | Zevia Zero Sugar Tea | B07GTYHFZF | Organic Stevia Leaf Extract | 12 oz Can | Zevia LLC |
|  | Saint James Iced Tea - Organic Black Tea | B0CDJDWD69 | Organic stevia leaf extract | 16 fl oz Bottle | Saint James Iced Tea |
|  | Liquid Death Iced Tea | B0DVTJD78K | Stevia Leaf Extract | 12 Fl Oz Bottle | Liquid Death |
|  | Saint James Iced Tea | B0CDJDWD69 | Organic Stevia Leaf Extract | 16 fl oz Bottle | Saint James Iced Tea |
|  | Ryl Iced Tea | B0BPN778TP | Stevia Leaf Extracts | 16 fl oz Bottle | The Ryl Co |
|  | Capri Sun Roarin' Waters | B01BSKPNBQ | Stevia Leaf Extract | 6 fl oz Pouch | Kraft Heinz |
|  | Marquis Natural Energy Drinks Sugar Free Zero Calories | B0C9P7JSRK | Organic Stevia Extract | 12 oz Can | Marquis |
|  | Swoon Half Unsweetened Iced Tea & Half Lemonade | B08V79PD5W | Stevia Leaf Extract | 12 Fl Oz cans | Swoon |
|  | Swoon Pink Lemonade | B08V6J38PC | Stevia Leaf Extract | 12 Fl Oz cans | Swoon |
|  | Swoon Swoonberry Lemonade | B0GKPM23RH | Stevia Leaf Extract | 12 Fl Oz cans | Swoon |
|  | Yerbaé Energy Beverage | B088NH4N2H | Organic Stevia leaf extract | 16 oz cans | Yerbaé |
|  | GORGIE Jordyn Jones Cherry Lime | B0DPL6RCYC | Reb M | 12 Fl Oz cans | Gorgie |
|  | Mateina Yerba Mate Zero Sugar Energy Drink | B0FF3PFM86 | Organic stevia extract | 12 fl oz Can | Mateina |
|  | Yerba Madre Organic Yerba Mate | B0C62QRW3Z | Stevia leaf extract | 15.5 fl Oz | Guayaki Sustainable Rainforest Products |
|  | GURU Lite Organic Energy Drinks | B001BOIVZA | organic stevia leaf extract | 12 fl oz Can | GURU Beverage Co. |
|  | Heywell Sparkling Adaptogenic Water Calm and Restore | B08VCRSQ4H | organic extract | 12 fl oz Can | KeHe Distributors |
|  | Liquid Death Sparkling Energy | B0FVB3JXKR | Reb A | 12 fl oz Can | Liquid Death |
|  | OLIPOP Minis Prebiotic Soda Pop | B0FBPMJVRD | Stevia Leaf | 7.5 fl oz Can | OLIPOP |
|  | Bloom Pop Prebiotic Soda | B0FJJXCFPL | Stevia Leaf Extract | 12 fl oz Can | Bloom Nutrition |
|  | Popwell Blackberry Lime Better | B0DV28PFCG | Stevia Sweetener | 12 fl oz Can | Talking Rain Beverage |
| Dietary Supplements | In The Raw Organic Stevia Sweetener Packets | B0FBCPMSJG | Organic Stevia Leaf Extract | 8.46 oz Sachet | Cumberland Packing Corporation |
|  | Whole Earth Stevia & Monk Fruit Liquid Sweetener | B07XC1SR3Q | Stevia leaf extracts | 1.62 Ounce Bottle | Whole Earth Sweetener Company |
|  | Enzo Easy Use Organic Stevia Powder | B0737DR48C | Stevia Extract | 200 g Bag | Enzo's Private Selection |
|  | SweetLeaf Sweet Drops | B013I4ESRU | organic stevia leaf extract | 2 Fl Oz Bottle | SWEET LEAF |
|  | Truvia Sweet Complete Zero | B0F8D19L6F | Stevia Leaf Extract | 32 oz Bag | Truvia |
|  | NOW Foods BetterStevia Organic Zero | B0017WG1J4 | Stevia Leaf Extract | 1 Pound Bag | NOW Foods |
|  | 365 by Whole Foods Market, Organic Stevia Powdered ExtraCount | B074H5Z5QZ | Organic Stevia Leaf Extract | 1 Ounce jar | 365 by Whole Foods Market |
|  | Life Enhancement InnerPower with Stevia | B07SLGZKKW | Reb A | 30 Servings bag | Life Enhancement |
|  | Micro Ingredients Pure Organic Stevia Powder | B0D2LYMLJT | Bitter Aftertaste Reduced Stevia Leaf Powder Organic | 1 Pound Bag | Micro Ingredients |
|  | Happy Belly Stevia Sweetener Powder | B0D183S46J | Stevia Leaf Extract | 9.8 oz Bottle | Amazon.com Services LLC |
|  | Stur Liquid Water Enhancer | B00D9K56RU | Stevia Leaf Extract (Reb A) | 1.62 fl oz Bottle | Stur |
|  | Pyure Organic Liquid Stevia Extract Sweetener | B072FJ47FF | Organic Stevia Leaf Extract | 1.8 fl oz Bottle | Pyure |
|  | HJUOOKD Stevia Extract Powder | B0GDFFWDJL | Stevia Leaf Extract | 7 oz bag | HJUOOKD |
|  | KAL Sure Stevia Extract Zero Calorie Sweetener | B007AU3HT4 | Stevia Leaf Extract | 8 oz Bottle | KAL |
| Functional Foods | Nongfu Spring Cha Pai Tea Drink | SC10633018211583 | Steviol glycosides | 500 ml/bottle | Nongfu Spring (Jiande) Xin’anjiang Drinking Water Co., Ltd. |
|  | Ambrosial AMX Sucrose-Free Black Diamond Yogurt | SC10534012100051 | Steviol glycosides | 200 g/bottle | Hefei Yili Dairy Co., Ltd. |
|  | Beijing Tongrentang Longyan Antihypertensive Tea | Health Food Approval No. (1999) 0231 | Stevia leaf | 2.5 g/bag | Anyang Longyan Sweet Tea Factory, Henan Province |
|  | Shengxiangyuan Steviol Glycosides | SC11337088100166 | Steviol glycosides | 1 kg/bag | Shandong Shengxiangyuan Biotechnology Co., Ltd. |
|  | Jianlibao Jinfang Yulu Botanical Beverage | SC10644060700170 | Stevia leaf powder | 500 ml/bottle | Guangdong Jianlibao Co., Ltd. |
|  | Xiangtang Zero-Calorie Sweetener | SC10633019901811 | Steviol glycosides | 500 g/bottle | Shandong Tangzhiwuyu Health Industry Co., Ltd. |
|  | Coca-Cola Sugar-Free Botanical Sweet Drink | SC10431011201303 | Steviol glycosides | 330 ml/bottle | Zhejiang Swire Coca-Cola Beverage Co., Ltd. |
|  | Miaoguniang Yigancao Sugar-Free Herbal Tea | SC10652272310072 | Stevia leaf | 310 ml/can | Guizhou Miaoguniang Food Co., Ltd. |
| Health Products | Beijing Tongrentang Longyan Antihypertensive Tea | Health Food Approval No. (1999) 0231 | Stevia leaf | 2.5 g/bag | Anyang Longyan Sweet Tea Factory, Henan Province |
|  | Baiyunshan Xingqun Xinxi Brand Sugar-Free Protein Powder for Middle-Aged and Elderly | SC12736098200238 | Steviol glycosides | 20 g/bag | Xinxi Biotechnology Co., Ltd. |
| Oral Care Products | Red Seal Fluoride Free Whitening Toothpaste | B0CSJX874H | stevioside | 3.53 oz/tube | Red Seal |
|  | Boka Fluoride Free Toothpaste | B0BSRFKKZJ | stevia leaf extract | 4 oz/tube | Boka |
|  | Dr. Sheffield’s The Original Toothpaste | B07CJ269WG | *Stevia Rebaudiana* Leaf/Stem Extract | 5 oz/tube | Dr. Sheffield's |
|  | Bentodent earthy natural toothpaste | B078JG16CF | Stevia Extract | 3.52 oz/tube | INDIAN DENTAL ORGANIZATION |
|  | Jieling Youkoujing Toothpaste | Guangdong Toothpaste Online Filing No. 2023101128 | Stevia Extract | 110 g/tube | Guangdong Xipai Daily Chemical Co., Ltd. |
|  | Kangaroo Mother Teenager Fresh Dental Care Toothpaste | Jiangsu Toothpaste Online Filing No. 2024000243 | Stevia Extract | 100 g/tube | Guangzhou Natural Kingdom Biotechnology Co., Ltd. |
|  | Sufangshe Plant Extract Whitening Toothpaste | Tianjin Toothpaste Online Filing No. 2025000360 | Stevia Extract | 100 g/tube | Tianjin Jikang Pharmaceutical Co., Ltd. |
|  | Koubao Jinzhi Jieyin Mouthwash | National Medicine Approval No. B20021009 | Steviol glycosides | 200 ml/bottle | Sichuan Enwei Pharmaceutical Co., Ltd. |
| Medicine | Jichuan Pharmaceutical Pudilan Xiaoyan Oral Liquid | National Medicine Approval No. Z20030095 | Steviol glycosides | 10 mL/vial | Jichuan Pharmaceutical Group Co., Ltd. |
|  | Jianwei Xiaoyan Granules (Sugar-Free Type) | National Medicine Approval No. Z32020496 | Stevia Extract | 5 g/bag | Suzhong Pharmaceutical Group Co., Ltd. |

**Note:** The table presents the use of stevia in different consumer goods, demonstrating its practical value and market prospects for low-sugar product development.
